# From the Soil to the Wine—Elements’ Migration in Monovarietal Bulgarian Wines

**DOI:** 10.3390/molecules30030475

**Published:** 2025-01-22

**Authors:** Elisaveta Mladenova, Tsvetomil Voyslavov, Ivan Bakardzhiyski, Irina Karadjova

**Affiliations:** 1 Faculty of Chemistry and Pharmacy, Sofia University, 1, James Bourchier Boulevard, 1164 Sofia, Bulgaria; voyslavov@abv.bg (T.V.); karadjova@chem.uni-sofia.bg (I.K.); 2 Technological Faculty, University of Food Technologies, 26, Maritza Boulevard, 4002 Plovdiv, Bulgaria; ivanbak81@yahoo.com

**Keywords:** soil, grapevine, wine, elements migration, correlation, FAES, ICP-OES, ICP-MS

## Abstract

Bulgarian wines are renowned worldwide and serve as a symbol of the country. However, ensuring wine authenticity and establishing reliable methods for its assessment are critical challenges in wine quality control. This study investigates the migration of chemical elements within the soil/grape/wine system and utilizes the findings to develop a method for identifying specific elements capable of distinguishing the geographical origin of wine. Additionally, it explores the potential to determine its botanical origin. Thirty monovarietal Bulgarian wines, specifically produced for this study with precisely known geographical and botanical origins, were analyzed for 20 chemical elements. These included macroelements such as Al, B, Ba, Ca, Cu, Fe, K, Mg, Mn, Na, P, Sr, and Zn, as well as microelements like As, Cd, Co, Cr, Li, Ni, and Pb. The study encompassed white wines from Chardonnay, Muscat Ottonel, Sauvignon Blanc, Tamyanka, and Viognier varieties, as well as red wines from Egiodola, Broad-Leaved Melnik, Cabernet, Cabernet Franc, Cabernet Sauvignon, Marselan, Melnik, Merlot, Pinot Noir, and Syrah. The chemical composition was determined in soil extracts (using acetate and EDTA extract to represent the bioavailable fraction), vine leaves, primary musts, and raw wines before clarification and stabilization. Statistically significant correlation coefficients were calculated for the soil/leaves, soil/must, and must/wine systems, enabling an analysis of the migration of chemical elements from soil to wine and the concentration changes throughout the process. The results identified elemental descriptors capable of indicating the geographical origin of wine.

## 1. Introduction

Bulgaria boasts a centuries-long tradition of winemaking. Wines produced under strict control of grape variety, regional origin, and production methods are highly esteemed for their originality and superior quality [1]. These wines derive their unique sensory characteristics from a blend of ecological and geographical factors, climatic conditions, specific production techniques, and the reputation of their producer [2]. They are crafted exclusively from grapes grown on designated terroirs, clearly identified on their labels, and processed following rigorously regulated methods under strict oversight throughout the vinification process [3].

In this context, authenticity and methods for detecting fraud are among the most critical concerns in wine quality control and safety. Research has led to the development of numerous methods to validate wine authenticity. One commonly used approach to determine the geographical/botanical origin of wine is mineral analysis.

The mineral content of wine is a critical determinant of its quality from several perspectives [4,5,6]. Key elements such as Fe, Cu, K, Ca, Zn, and Al significantly enhance wine’s health benefits and overall quality [7,8]. Conversely, toxic elements like As, Cd, Pb, and Hg pose health risks to consumers [9]. Additionally, specific elements serve as descriptors for assessing the geographical and botanical identity of wine [10].

The International Organization of Vine and Wine (OIV) regularly updates the methodologies for monitoring the mineral content of wines and grape juice, ensuring precise and reliable assessments [11].

Modern methods enable precise and reliable determination of the mineral and isotopic profiles of wines, grapes, and soils from specific grape-growing regions [12,13,14]. Various instrumental techniques are employed to measure selected elements in extracts, mineralized grapevine parts, and wine samples such as inductively coupled plasma mass spectrometry (ICP-MS) and isotope ratio mass spectrometry (IRMS), which are leading technologies due to their high sensitivity, selectivity, throughput, and capability to analyze multiple elements simultaneously [14,15,16,17]. The concept to quantify as much as possible chemical elements usually uses combination of methods, including electrothermal atomic absorption spectrometry (ETAAS) [18,19,20], cold-vapor atomic absorption spectrometry (CV-AAS) [21], cold-vapor atomic fluorescence spectrometry (CV-AFS) [22], and inductively coupled plasma optical emission spectrometry (ICP-OES) [17,23].

Numerous studies have supported the use of mineral composition as an effective tool for identifying the geographical origin of wines [15,24,25]. This concept is based on the hypothesis of a strong correlation between the soil’s elemental profile and the chemical element composition of wine [23,26,27]. It is widely accepted that the mineral content of wine reflects the soil composition of the vineyard, as soil is the primary source of chemical elements for the grapevine [28]. However, contributions from aerosol deposition should also be considered [29]. While soil provides the essential nutrient supply, the bioavailability and uptake of chemical elements are largely determined by the grapevine’s physiological and biochemical needs through its root system [30,31]. Various macroelements, such as potassium and calcium, are absorbed from the soil, where potassium accumulates in grape tissues and calcium supports overall plant growth and development. Other elements, including sodium, magnesium, and trace elements like iron, copper, zinc, cobalt, and manganese, influence enzymatic and biological processes in the grapevine [32]. Notably, the solid parts of grape berries—such as skins, cell walls, pulp, and seeds—tend to contain higher concentrations of these elements [23]. The literature showed that each grape variety exhibits a unique elemental profile, reflecting its distinct capacity for element absorption. This individual “elemental image” results from the varying nature of element uptake by different grape varieties. It is worth to take into account that the chemical composition of plants is largely influenced by the elemental composition of the soil, though it does not directly mirror it [33]. The issue of “contamination” in vineyard soils is increasingly relevant due to anthropogenic factors, such as the use of fertilizers [34], irrigation water [35], and chemical sprays [36]. Organic and mineral fertilizers applied to vineyards can have varying impacts on the chemical composition and quality of both the grapes and the resulting wine [37]. Tracking the migration of elements from soil to wine, some researchers analyze the total elemental content in soil, grapes, and wine [14]. Single-extraction procedures, commonly employing extractants such as Na_2_EDTA, NH_4_NO_3_, CaCl_2_, and deionized H_2_O, are widely recommended for studying the bioavailability of major and trace elements in agricultural soils [38]. Among the extractants, Na_2_EDTA has been identified as the most effective for extracting elements such as Al, As, Ca, Cd, Co, Cu, Fe, K, Mn, Pb, V, Sr, and Zn. Acetic acid (CH_3_COOH) is selective for extracting elements like B, Be, Cr, Li, and Ni, while NH_4_NO_3_ is particularly suited for Ba and Sr. In contrast, weak salt solutions such as CaCl_2_ and deionized water are less aggressive and extract fewer elements [39]. For instance, high potassium levels in vineyard soils can result in elevated potassium concentrations in grape juice, leading to increased pH values [40].

The chemical composition of final wines is influenced not only by the grape and soil but also by the vinification process. During fermentation and aging, the concentrations of certain elements can change due to enzymatic activity and precipitation reactions [41]. To clarify raw wine and enhance the final product’s quality, bentonites are commonly used, leading to a redistribution of chemical elements [42].

The chemical composition of wine is influenced by numerous factors that are challenging to fully quantify and control. On one hand, the ratios of mineral nutrients in the final wine exhibit a complex, indirect, and distant correlation with the geological minerals present in the vineyard. On the other hand, the bioavailability and uptake of these minerals are influenced by plant-specific characteristics and climatic conditions. Consequently, detecting wine adulteration and determining its regional origin represent intricate analytical challenges. The aim of this study is to evaluate the extent to which the chemical element composition of wine reflects the mineral content of the soil. The research aims to establish how soil profiles influence the mineral composition of vine leaves, primary must, and raw wine (prior to clarification and stabilization). To determine the bioavailable concentrations of elements in soil, two extraction methods were employed: 0.43 mol/L acetic acid and 0.05 mol/L EDTA. The study monitored changes in the concentrations of both essential and non-essential elements throughout the wine production process and assessed the bioaccumulation of elements in two above-ground parts of the vine—leaves and grapes (presented as primary must). Based on the results, an approach has been proposed to trace the migration of macroelements such as Al, B, Ba, Ca, Cu, Fe, K, Mg, Mn, Na, P, Sr, and Zn, alongside microelements like As, Cd, Co, Cr, Li, Ni, and Pb. This method aims to identify significant descriptors for wine composition. We conclude that combining changes in the mineral content across the soil–vine–wine system with Pearson correlation coefficients provides an effective strategy for selecting an optimal set of elements to determine the geographical origin of wine. Additionally, the study explores the potential to evaluate the botanical origin of wine, presenting conclusions on this aspect.

## 2. Results

### 2.1. Elements’ Content in Soil, Leave, Must and Wine

Tables S1–S4 present the full dataset for 13 macroelements’ content (Al, B, Ba, Ca, Cu, Fe, K, Mg, Mn, Na, P, Sr, and Zn) and 7 microelements’ content (As, Cd, Co, Cr, Li, Ni, and Pb) in the analyzed samples—soil extract (CH_3_COOH and EDTA), grapevine leaves, must, and wine (red and white). The mean and median values are also presented. As can be seen from Tables S1 and S2, the mean and median values for elements Ba and K in soils and leaves are very different. This means that these elements’ content is in large variance due to different sampling regions and is not in accordance with the grape’s variety (red or white). The same is observed for microelements like Li and Pb (see Tables S3 and S4). Therefore, we do not present any standard deviation values for chemical elements’ content in analyzed samples.

### 2.2. Correlations in the System Soil/Soil

In order to identify correlation between elements’ content in the system soil/leaves, soil/must, and must/wine, a Pearson correlation analysis and linear fitting were performed. Results for the calculated correlation coefficients are presented in Table S5.

**Figure 1:**
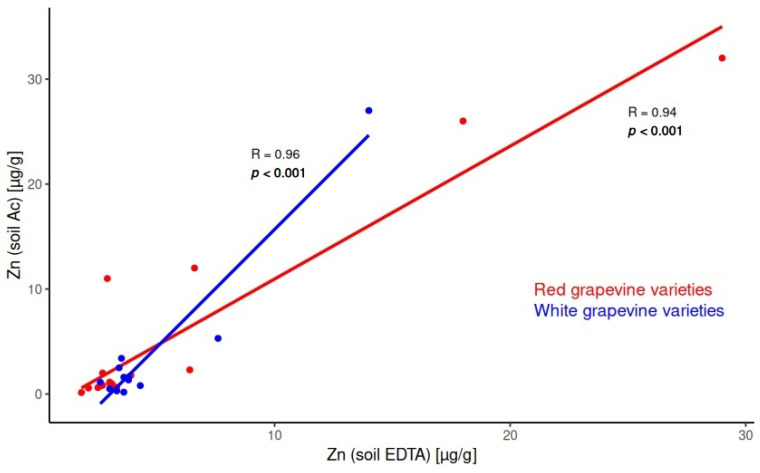
Significant correlation for Zn content in soil between the acetic acid and EDTA extracts in red and white grapevine varieties.

According to the obtained results, a significant correlation between the acetic acid and EDTA soil extracts can be observed for 10 macroelements. Figure 1 shows the positive correlation for Zn. The same curve and desired Pearson’s coefficients (*p* < 0.05) are also observed for elements Al, B, Ba, Ca, Cu, Fe, K, P, and Sr. Similar models were obtained for both red and white grapevine varieties.

**Figure 2:**
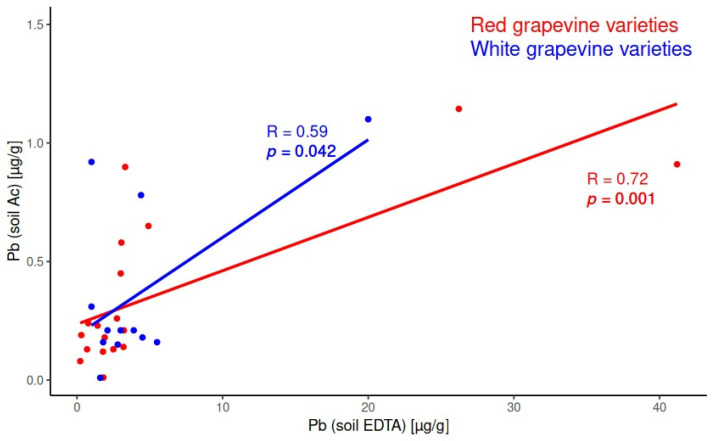
Significant correlation for Pb content in soil between the acetic acid and EDTA extracts in red and white grapevine varieties.

Significant positive correlations for microelements’ content between acetic acid and EDTA soil extracts were obtained for As, Cd, Co, Li, Ni, and Pb for red grapevine varieties and for Li and Pb for white grapevine varieties. Figure 2 presents the linear fitting and statistically significant Pearson coefficients for the system acetic acid soil extract/EDTA soil extract for Pb for red and white grapevine varieties.

### 2.3. Correlations in the System Soil/Must

**Figure 3:**
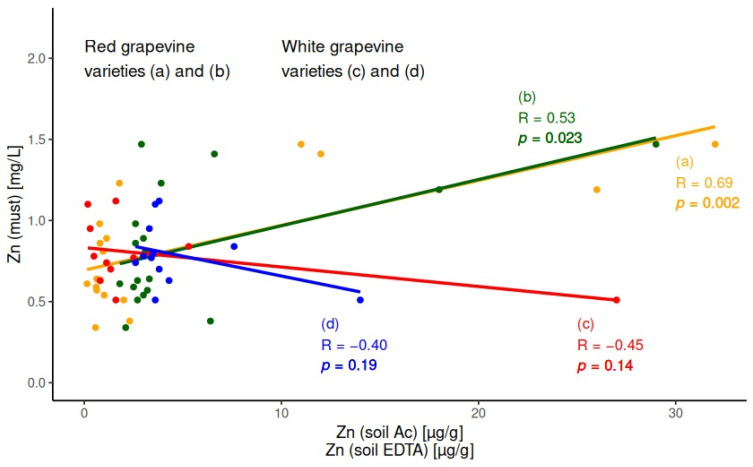
Correlations for Zn between: (a) acetic acid soil extract and must for red grapevine varieties; (b) EDTA soil extract and must for red grapevine varieties; (c) acetic acid soil extract and must for white grapevine varieties; (d) EDTA soil extract and must for white grapevine varieties.

A significant positive correlation between soil extract (as for acetic acid, as for EDTA) and must samples was obtained for Zn only for red grapevine varieties. As can be seen in the figure, the correlation coefficients of linear fittings (a) and (b) are R = 0.69 and R = 0.53, and the calculated probability values are *p* = 0.002 and *p* = 0.023 for acetic acid extract and EDTA extract, respectively. No significant correlations for Zn were obtained for white grapevine varieties between the same pairs—acetic acid soil extract/must and EDTA soil extract/must. The models are presented graphically in Figure 3 through linear fittings (c) and (d). The *p* values (*p* = 0.14 and *p* = 0.19, respectively) confirmed the null hypothesis and show that there is no significant correlation.

For all determined elements (without any exceptions), it is observed a decrease in their content from must to wine samples. Therefore, no correlation coefficients and linear fitting curves are presented for the system must/wine.

### 2.4. Elements’ Decreasing Level in the System Must—Wine

Chemical elements’ content was found to decrease from must to wine samples. The levels of their decreasing (*D*%), expressed as a range from minimal to maximal value, are presented in Table 1 and Table 2 for macroelements and microelements content, respectively.

## 3. Discussion

The results presented in Table S1 show that in the leaves the highest concentrations were observed for the essential elements Ca, K, and Mg and even more for the elements K, P, and Mg; these concentrations were higher than those in the soil extracts. The behavior of the microelements Zn, Fe, Cu, and B was found to be similar with concentrations in the leaves—higher than that in the soil extracts. The results for wines are significantly different—much lower than those in the leaves, the highest levels were found for K, followed by the concentration for P. The concentration levels obtained for chemical elements in leaves and wine are in good agreement with the literature data.

### 3.1. Soil Characterization Based on Soil Extracts

Soil is an intricate three-phase system—comprising solid, liquid, and gaseous components—where chemical elements exist in various forms and engage in diverse processes, such as phase distribution, adsorption–desorption, complex formation, and precipitation. These processes result in significant spatial and temporal variability of chemical elements. Furthermore, the inclusion of a fourth phase, the living biotic component, makes the system even more complex. In this dynamic system, the fraction of metals presented in the soil solution, which can be easily released under specific environmental conditions within the rhizosphere, is generally considered available for plant uptake.

To estimate the bioavailability of metals, a variety of chemical extraction methods have been developed. However, no single extractant has been universally accepted as the most effective or representative of real-world conditions. In this study, two single-step extraction procedures—0.43 mol/L acetic acid and 0.05 mol/L EDTA—recommended by the Community Bureau of Reference (BCR) of the European Commission were employed to characterize the bioavailability of chemical elements in soil samples. These extractants are understood to target different fractions of soil-bound elements. Acetic acid primarily extracts metals adsorbed on soil clay minerals via electrostatic interactions or present in carbonate forms [43], while EDTA captures elements bound in chelate complexes within the soil solution [44]. This dual approach provides insights into the availability of various chemical species under differing soil conditions. This study focused on soils from various wine-growing regions of Bulgaria to investigate how their chemical element content influences the composition of wines prior to clarification processes. To ensure representation, at least two wines from different grape varieties were selected for each soil type.

The results showed a correlation between the two extracts for elements more readily released due to desorption and competitive complexation reactions. For essential elements like Cu, Fe, and Zn, EDTA extracts exhibited significantly higher concentrations than acetic acid extracts. This disparity is likely because plants in the rhizosphere release ligands that enhance the bioavailability of these elements, particularly for Fe, which is otherwise insoluble at soil pH values above 5.

Key nutrients for plants, including grapevines, are nitrogen, phosphorus, and potassium, followed by essential elements like Ca, Mg, and S, as well as trace elements such as B, Cu, Fe, Mn, and Zn. Notably, soils from the Oryahovo and Brestnik regions were identified as calcareous, with high Ca content. The iron content varied significantly across the sampled soils, with sub-regions such as Starosel, Topoli Dol, and Chernodab exhibiting statistically higher Fe concentrations.

Data from Tables S1 and S2 highlight regional variations in the element distributions. For example, soils in the Oryahovo and Pirgovo sub-regions (Danubian Plain, locations (1) and (2) in Figure 1) had lower K content but higher Na levels. Conversely, soils from the Starosel, Topoli Dol, Chernodab, Levunovo, General Todorov, and Vranya sub-regions (Rose Valley, Thracian Valley, and Struma Valley in Central and Southern Bulgaria) showed lower Ca and Mg levels but higher Ba concentrations.

Toxic microelements were detected at levels well below the national permissible limits for agricultural soils. Pearson correlation coefficients indicated significant positive correlations between the concentrations of essential elements in both soil extracts across vineyards with red and white grape varieties. Positive correlations for microelements were also observed: for As, Cd, Co, Li, Ni, and Pb in red grape vineyards and for Li and Pb in white grape vineyards.

Aluminum chemistry in soils was found to be particularly complex, involving diverse organometallic and multinucleated complexes. Acetic acid proved more efficient for Al extraction compared to EDTA, as reflected in the data from Tables S1 and S2. This research emphasizes the nuanced relationships between soil composition and wine chemistry, highlighting the importance of bioavailable elements and their interactions in soil systems.

### 3.2. Chemical Elements Migration in the System Soil/Plant

The study on the metabolism of elements in plants highlights that the chemical profile of a plant depends not only on the soil environment but also on the plant’s physiological processes. This underscores the importance of investigating each soil/plant system individually. The migration and distribution of chemical elements in plants also vary depending on the plant’s morphology, which results in different concentrations of elements in different parts of the plant. While the grape is the primary focus for assessing the chemical profile of wine, this study also examines the content of elements in the leaves and primary must (the initial grape juice before fermentation), which is fermented to produce wine.

The values of correlation coefficients presented in Table S5 are almost identical independently of the extractant used. The study found significant differences in the correlation coefficients between bioavailable concentrations in soils and concentrations in leaves for white and red wine varieties. This is in line with the conclusions of [45] that grapevine varieties influence chemical elements bioaccumulation in leaves. However, the elements with significant different behavior indicating grape variety in our study differ from the elements in the study [45]. For red wines, a positive correlation was observed for essential elements like B, Mg, and Zn, whereas for white varieties, only Zn showed a positive correlation. The study also found no correlation between the primary macroelements K and P in either grape variety. However, a positive correlation was observed for the elements Ba and Sr in both red and white wine varieties. Among the microelements, only Pb showed a positive correlation for both wine types.

The lack of correlation between K and P suggests that grapevines may have regulatory mechanisms that enable them to uptake the necessary levels of these elements independently of soil content, ensuring optimal plant growth. This was further supported by the narrow range of K and P content found in the leaves, as indicated by the similar mean and median values.

The geographical locations of the sub-regions, particularly those near water bodies, may also explain the lack of correlation for sodium. For instance, the Oryahovo sub-region, near the Danube River, and the Pirgovo sub-region, near the Black Sea, may experience different Na concentrations in the soil, influencing plant uptake.

As expected, Fe leaching was more effectively achieved using the EDTA extraction method, with higher concentrations observed in certain sub-regions such as Starosel, Topoli Dol, and Chernodab. However, this did not correlate with higher iron levels in the leaves from these sub-regions, suggesting that other factors influence iron uptake in the grapevine. The critical iron content for grapevine leaves is typically in the range of 50–150 µg/g, and most of the samples analyzed in this study met this requirement, indicating adequate iron availability for plant growth.

This study reinforces the complexity of the soil/plant/wine system and suggests that various environmental, physiological, and regional factors play significant roles in determining the mineral composition of wine.

Manganese is a critical micronutrient for plants and can impair growth when present in either limiting or excess quantities [46]. As can be seen in Tables S1 and S2, the Mn content in analyzed soils and leave samples varied in a wide concentration range and were higher in the acetic acid extract. Dissolved Mn(II) in a soil solution is moved through roots and transported primarily to leaves [47]. Manganese accumulates in leaves as aqueous or organic-bound Mn(II) and is not translocated to other plant tissues or reabsorbed during senescence [48]. However, as mentioned, a positive correlation between Mn concentrations in leaves and concentrations in soil extract was found only for white wines with extract in EDTA.

Calcium and magnesium are important for the physiology of the grapevine. Plants utilize Ca^2+^ to strengthen cell walls, neutralize vacuolar anions, and provide stress protection [49]. Cellular Mg^2+^ is highly abundant and acts as a cofactor for a wide range of enzymes and serves as the core metal of chlorophyll molecules in green tissues [50]. Several families of ion channels and transporters have been identified that contribute to Ca^2+^ and Mg^2+^ transport across plasma membrane and intracellular membranes [51]. That is why high Ca content in soil extract reflects their high content in leaves for red varieties. The other alkaline earth elements (Ba and Sr) have similar chemical behavior as Ca and probably used the same transporter channels. This probably explains the positive significant correlation found for Ba, Ca, Mg, and Sr for the examined system soil/leaves.

The chemistry of Al interactions in soils is remarkably complex and still not fully understood by researchers, possibly due to the wide array of organometallic and multinucleated complexes and co-occurring ions in soils [52]. As can be seen from the results presented in Tables S1 and S2 independent of its various species presence in soil samples, Al content was stabilized in leaves, must, and wine samples. This explains that the plant (grapevine) has a strong and excellent bioregulatory mechanism for Al uptake independent of grape variety and soil location.

The behavior of Cu is governed by its essentiality from one side and relatively frequently application of Cu-fungicides in viticulture to control downy mildew [53]. The Cu concentration was found to be highest in the leaves than in grapes. The risk-based limit of Cu for soil used for food crop production is 200 µg/g [54]. As can be seen in Tables S1 and S2, Cu was mainly leached with EDTA extraction procedure. This means that Cu is presented in analyzed soils mostly as a chelate complex bound to organic matter. And the highest concentration obtained measured was 95 µg/g—far away from the critical value reported. The average Cu content generally in the plant dry weight has been approximately recorded at 10 µg/g [55]. In typical grapevine leaves, the Cu content is normally 30–35 µg/g [24]. Grapevines generally thrive at about 35 µg/g Cu but can tolerate up to 148 µg/g [56]. The results obtained in the frame of this project are in great agreement with reported values. As presented in Tables S1 and S2, the Cu content in leaves samples varied in wide range 1.50–216 µg/g and mostly depended on environmental conditions. The highest contents were observed for the Oryahovo sub-region (see Figure 1, (1)). This vineyard area is located just above the water of the Danube river. In such atmospheric conditions, a vineyards’ treatment with Cu-fungicide is normally significantly more often (till 12 times per vintage year). This explained the lack of correlation between Cu concentrations in leaves and soils.

The elements Zn (essential element) and Pb (toxic element) exhibited similar behavior for all studied varieties. They yielded a significant positive correlation between both soil extracts, between soil and leaves, and even between soil and must. Most probably, both elements are complexed with soil organic matter (humic, fulvic acids, etc.). The stability constant of Pb with humic acid was greater than the stability constant of Zn with humic acid. The decreasing trend was determined as Pb > Ca > Cd > Zn > Mn > Mg. [57]. This is probably the reason why Pb uptake from grapevine leaves was significant and positively correlated with soil content. Most probably, Pb is transported through the biological pathways of essential elements’ uptake.

The concentrations of elements in the primary must do not directly mirror the concentrations found in the grapes, as the winemaking process influences their distribution. For example, in red wines, the grapes are opened, and the surrounding leaves are removed before fermentation, which exposes them to the environment for a longer period. In white wines, the grapes remain concealed behind leaves during ripening, resulting in a shorter time in the initial mixture before fermentation. Therefore, the primary must better represent the soil profile’s influence on the final wine product.

As might be expected, the correlations between elements concentrations in the primary must and soil extracts are different for red and white varieties due to the different process of primary must preparation. A positive correlation was found for the essential macro elements Ca, Fe, and Zn in the red varieties and for no one of essential elements in the white varieties. Evidently, elements with characteristic concentrations in grape skin determine such positive correlations for red varieties. For nonessential elements, positive correlations for both red and white varieties were observed for Sr. Positive correlations for microelements were found for Cd, Li, and Pb for the red varieties and no correlations were found for microelements in the white wines most probably due to the same reason—primary must preparation.

Manganese accumulates mostly in leaves and presents in low concentration levels in must and wine samples analyzed—0.6 to 2.7 mg/L in must and 0.4 to 1.9 mg/L in wine samples. No positive correlation was found for Mn neither in the red nor in the white wines.

For Zn, a positive correlation was observed only for red grapevine varieties but not for white ones. An additional explanation of Zn’s correlation dependencies is that Zn accumulates mainly in grape skin and seeds. These grape’s parts participate in the alcoholic fermentation process for red wines. In white wines, the technology fermentation process includes only the must juice, without grape skins and seeds. These parts have contact with grape juice for only a couple of hours before starting fermentation. This is an assumption for Zn to be leached from grapes and present in the future wine product.

The same relationship was observed for K—a significant positive correlation for the system soil/must, especially for red grapevine varieties. Similar to Zn relationships, this is due to high K contents in grape bunches, skin, and seeds.

No statistically significant correlation between the concentrations of alkali earth elements (Ca and Mg) in the soil extracts or must was found except for Sr. This confirms the idea that plants (e.g., grapevines) undergo smart bioregulation uptake processes in order to ensure their normal physiology.

### 3.3. Chemical Elements in the System Must/Wine

The winemaking process, especially the stages of grape processing, fermentation, and skin maceration, significantly influences the mineral content of the final wine. These processes are key in altering the concentrations of both essential and nonessential elements, which can affect both the quality and sensory characteristics of the wine.

One of the most important phases for mineral changes is fermentation. During this stage, the concentrations of several elements—such as Al, Cd, Co, Cr, Cu, Fe, Mn, Pb, and Zn—tend to decrease. These reductions are typically associated with their chemical properties, enzymatic consumption, and the formation of precipitates during fermentation. The enzymes involved in fermentation play a crucial role in the consumption of certain elements, particularly those that are essential for yeast growth and metabolism, such as Ca, Cu, Fe, K, and Zn [58].

As indicated in the results, a statistically significant decrease in the concentrations of these elements occurred after fermentation. For the red wines, elements like Al, Ba, Cu, Fe, Na, Sr, and Zn showed reductions of over 70% in some cases. This is consistent with previous studies that have highlighted the significant decrease in several elements during fermentation [59,60]. The reductions in white wines were also significant, particularly for elements like Al, Ba, Cu, Mn, and Na.

For Ca and K, decreases in concentration were observed across both red and white wines, but the red wines typically exhibited higher reductions. This is likely due to the precipitation of these elements as tartrate (for K) and tannate (for Ca) during fermentation. In the white wines, precipitation processes still occurred, but the extent of the decrease was somewhat lower, with reductions in the range of 20–60% for both calcium and potassium.

These changes suggest that the winemaking process, through fermentation and precipitation reactions, plays a critical role in shaping the mineral profile of wine. The decrease in certain elements can be linked to their removal or transformation during fermentation, as well as their interactions with other compounds in the wine, such as proteins, acids, and phenolic compounds. The extent to which these elements are reduced also depends on the chemical nature of the element and its affinity for precipitation during fermentation.

Overall, the results highlight the importance of fermentation and maceration in determining the final mineral composition of wine, which can affect both its flavor profile and its nutritional content.

The study reveals several important insights regarding the relationship between essential macroelements and microelements in red and white wines. For essential macroelements, positive correlations were found between the concentrations of B, K, Mg, P, Sr, and Zn in red wines, while for white wines, positive correlations were observed for B, Fe, P, and Zn. These elements play vital roles in various biochemical processes, including enzyme activity, cell division, biomass production, and the breakdown of carbohydrates and amino acids in both the plant and fermentation stages.

The absorption of mineral ions by yeast cells and their subsequent physiological functions have been well studied. However, their specific roles in fermentation and flavor production remain somewhat unclear, as the fermentation environment is complex and influenced by various factors. For example, while the general understanding of yeast metabolism and the importance of elements like magnesium and zinc is well established, their direct contribution to the flavors of the final wine remains less well defined. The mineral profile of wine, particularly in relation to fermentation, could affect aspects like yeast activity, fermentation kinetics, and the development of aroma compounds, which contribute to the final sensory characteristics of the wine.

In addition to the essential elements, significant correlations were found for the nonessential elements Mn and Sr in both red and white wines, with a correlation for Al found specifically in white wines. The changes in the concentrations of these microelements are likely connected to their chemical behavior during fermentation. For instance, the precipitation of certain elements, like Fe and Zn, may occur due to interactions with wine proteins, which could explain their decreased concentrations in the final wine.

The study found a pronounced decrease in the concentrations of microelements in the wines, with the levels of many elements dropping by approximately 70% or more. This significant reduction in microelements during the winemaking process is likely due to various chemical reactions, including precipitation, complexation, and adsorption processes, which are particularly prominent during fermentation and post-fermentation steps. Some elements, such as As, Co, and Ni, did not exhibit positive correlations in the study, possibly due to their limited bioavailability or their tendency to form stable, insoluble compounds that do not participate actively in the fermentation process.

Overall, these findings suggest that the mineral composition of wine is shaped not only by the soil and vine but also by the complex biochemical processes occurring during fermentation, which can influence both the nutritional profile and sensory properties of the final product.

Essential elements such as B, Fe, K, Mn, Na, and P play vital roles in various biological processes during fermentation. These processes include enzyme activity, yeast metabolism, and the overall fermentation dynamics that transform the must into wine. Due to their crucial biological functions, the concentrations of these elements in the system (must to wine) are largely independent of the grape variety and geographical region. This suggests that the biological processes governing their uptake and utilization during fermentation are universal, regardless of the environmental or varietal conditions.

In contrast, the behavior of potentially toxic elements, such as As, Cd, Cr, Ni, and Pb, was found to be different. The recorded concentrations of these elements in the analyzed wines do not exceed the maximum permissible limits set by the International Organization of Vine and Wine (OIV), ensuring that the wines are safe for consumption [11]. As mentioned, the concentrations of these toxic elements decrease significantly throughout the must-to-wine process. This reduction could be attributed to several factors, including their transformation into chemical species such as chelate complexes.

Previous studies have shown that yeast, which is present in grape skins, has the ability to bind toxic metals and remove them from the aqueous solution during fermentation [61]. This mechanism might explain the observed decrease in the concentrations of toxic elements, as the yeast cells effectively sequester these elements, reducing their presence in the final wine product.

The decrease in toxic elements during fermentation may also be influenced by their chemical interactions with compounds in the grape skins, such as phenolics and organic acids, which can form complexes with metals. These interactions could lead to the precipitation or binding of metals, reducing their bioavailability and thus their concentration in the final wine.

Overall, the reduction of both essential and toxic elements during the must-to-wine process highlights the complex interactions between the grapes, yeast, and other compounds in the wine matrix, all of which contribute to the final mineral composition of the wine.

## 4. Materials and Methods

### 4.1. Sample Collection

#### 4.1.1. Sampling Points

This paper is a part of a larger project aimed to qualitatively and quantitatively characterize Bulgarian monovarietal wines. For this purpose, vineyards between 6 and 10 years old were included in the research. At that time, the vine is in an active fruiting period, wherein there is a balance of metabolic processes in the plant in terms of fruiting and grape composition. The vineyards for sampling are under exploitation from qualified wineries. Regarding this, all of the obtained samples had geographically known origins, the vineyards were grown in accordance with national regulations, and the obtained must and row wine were of proven monovarietal origin.

**Figure 4:**
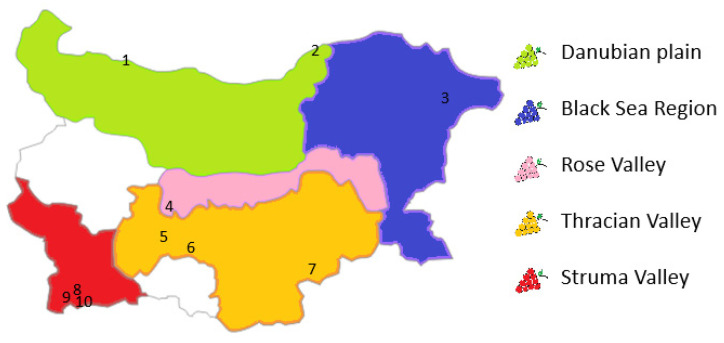
Division of territory of Bulgaria to five wine-growing regions. The numbers show the sampling sub-regions, which are listed in Table 3.

As can be seen from Figure 4, the territory of Bulgaria is divided to five wine-growing regions. The Arabic numbers on the map show the sub-regions that locate the sampling places in the range of this project.

Several vineyards are grown in some of the sampling sub-regions—some of them with different grape varieties and some of them with one grape variety. In Table 3 are presented the grapevine varieties, which were collected from each sampling sub-region in order to obtain 30 samples. From each of the 30 sampling places, soil samples were collected from the vineyard area, as well as leaves from the grapevine and must samples, which were obtained using the grapes from the sampling vineyard and row wine, which was produced after fermentation of the previous must. The whole sampling process was carried out in 2019.

#### 4.1.2. Soils

Soil samples were collected from 6 different locations of each studied vineyard in an amount of 500 g, from the rhizosphere region, at a depth of 40–50 cm. In this soil layer is that part of the vine root system most actively involved in the absorption of elements from the soil that thus nourishes the vine. The soils from each vineyard were homogenized and dried at room temperature for one week. The soil samples were sieved through a 2 mm sieve. The soil samples prepared were kept in paper bags in a dry, cool, and well-ventilated place till the time of analysis.

#### 4.1.3. Leaves

Leaves samples were collected from healthy, normally developed (typical) vines of the given variety near the places where a soil sample had been taken. In order to look for a direct relationship between chemical elements content in soil, leaves, grapes, must and wine, leaves samples were collected during the grape harvesting. From each sampling grapevine, an amount of 10–15 leaves was collected from different heights of the shoots. The leaves were dried under atmospheric conditions at a temperature of 30–40 °C for 5 to 7 days. After that, all samples of leaves were kept in paper bags in a dry, cool and well-ventilated place till the time of analysis.

#### 4.1.4. Must

Approximately 50 kg of grape sample in a technological maturity was collected from each grape variety, vineyard of sub-region, and region according to data in Table 3. The must preparation technology included the following: sorting; destemming; crushing; obtaining a must self-flow, which was near 40–45% from the weight of manufactured grapes. For white grape varieties, the sugar content at which the harvest was carried out varied between 21 and 23% (*w*/*v*). The lower values 21–22% (*w*/*v*) apply to Muscat Ottonel, and the higher 22–23% (*w*/*v*) apply to Tamyanka, Sauvignon Blanc, Viognier, and Chardonnay. For red grape varieties, the sugar content varied around 23–24% (*w*/*v*). Soil and leave samples were collected at the same time. Must sulfidation was carried out with 100 mg/L SO_2_ and cold settling by temperature around 10 °C. A volume of 5 mL of prepared must was preserved in a glass tube with 5 mL suprapur concentrated HNO_3_ (65%, Sigma-Aldrich, CO Wicklow, Ireland) and kept in fridge until analysis.

#### 4.1.5. Row Wine

The grapes from which the must samples were obtained were vinified in the certified wineries until separate batches of wine were obtained. The technology for white wines production included alcoholic fermentation at musts’ typical temperatures of 14–16 °C. The technology for the production of red wines included alcoholic fermentation of grape marcs at temperatures of 26–28 °C.

In both cases, yeast strains of the genus *Saccharomyces cerevisiae* were used in a lyophilized state, suitable for the respective grape varieties. Before their introduction into the process, they were rehydrated according to methods recommended by the producers.

Upon reaching a relative density of 1.000 g/cm^3^, the young red wines were separated from the solids and further fermented separately from them. The end of alcoholic fermentation of white and red wines was established by chemical analysis for reducing sugars according to method OIV-MA-AS311-01A [62]. When the reducing sugar values remained unchanged in 3 consecutive analyses, alcoholic fermentation was considered to be complete. Finally, white wines were treated using 40 mg/L free sulfur dioxide. Red wines were inoculated with a pure culture of lactic acid bacteria *Oenococcus oeni* (again in lyophilized form). The progress of malolactic fermentation was monitored by paper chromatography. After the disappearance of the malic acid spot, fermentation was considered to be complete, and samples were sulfated using 40 mg/L free sulfur dioxide followed.

Five days after the addition of free sulfur dioxide young wines (white and red) were raked from the lees and stored in a full tank. Where this was not possible, the empty space in the tank was saturated with inert nitrogen gas.

The young wines were stored for 6 months for self-clarification at a cellar temperature between 12 and 15 °C.

They were not subjected to any forced clarification or stabilization treatments, because according to our research [42], these treatments strongly modify the mineral compositions of wines.

### 4.2. Sample Pretreatment

Three parallel samples were analyzed for each type of sample (soil, leave, must, wine). A blank sample was prepared and analyzed according to the analytical procedure used.

#### 4.2.1. Soil Samples

In the frame of this study, bioavailable concentrations of elements in soil were determined by using two single extracts using 0.43 mol/L CH_3_COOH (pH = 3.40) and 0.05 mol/L EDTA (used as ammonium salt, pH = 7) [63,64]. Concentrated acetic acid for analysis ≥96% (Emsure, Merck, Darmstadt, Germany) was used for the preparation of 0.43 mol/L CH_3_COOH by appropriate dilution with Milli-Q water (Millipore purification system Synergy, Darmstadt, Germany). The EDTA ammonium salt was prepared from EDTA-free acid (ACS reagent, Sigma-Aldrich, Oakville, ON, Canada) and concentrated ammonia solution (28–30%, Emsure, Merck, Darmstadt, Germany) after appropriate dilution with Milli-Q water. Orbital shaker OS-20 (Boeco, Hamburg, Germany) and Hettich Zentrifugen EBA 200 (DJB Labcare, Newport Pagnell, UK) were used.

#### 4.2.2. Leaf Samples

The leaf samples were grinded to a fine powder. About 0.5 g leave powder sample was accurately weighed into a small clean beaker, 5 mL conc. HNO_3_ (≥69.0% TraceSELECT, Honeywell, Fluka, Seelze, Germany) was added, and the mixture was left to stay under a watch glass overnight. After that, the sample was slowly and carefully heated on a sand bath for about 4 h until the sample was almost completely digested and the acid was completely fumed. After cooling, the dry residue was quantitatively transferred into a clean container using deionized water to an accurate weight of about 20 g. The solution was filtered through a syringe filter with a membrane pore size of 0.25 µm.

#### 4.2.3. Must Samples

The must samples (preserved with conc. HNO_3_) were quantitatively transferred in 50 mL beakers and carefully heated on a sand bath until must digestion. The excess acid was fumed, and samples were quantitatively transferred in clean containers with deionized water to an accurate weight of 12 g. The solutions were filtered through a syringe filter with a membrane pore size of 0.25 µm.

#### 4.2.4. Wine Samples

About 20 g wine samples were accurately weighed in 50 mL beakers. The samples were evaporated slowly and carefully on a sand bath almost to dryness. After that, about 3 mL of conc. HNO_3_ was added to the warm mixtures, beakers were covered with watch glasses, and the samples were slowly digested for about 1 h. At the end, the solution was carefully fumed, and the samples were quantitatively transferred in clean polypropylene centrifuge tubes to an accurate weight of 20 g. The solutions were filtered through a syringe filter with a membrane pore size of 0.25 µm.

### 4.3. Instrumental Measurement

The concentrations of 12 elements (Al, Ba, Ca, Cr, Cu, Fe, Mg, Mn, Ni, P, Sr, and Zn) in soil extracts, digested leaves, must, and wine samples were measured by inductively coupled plasma optical emission spectrometry (ICP-OES, Ultima 2, Jobin Yvon Horiba, Edison, NJ, USA) under optimal instrumental parameters [65]. Stock multielement standard solution of 1000 mg/L was used for the preparation of working standard solutions of Al, Ba, Ca, Cr, Cu, Fe, Mg, Mn, Ni, Sr, and Zn (ICP Multielement Standard Solution IV, CertiPUR, Supelco, Bellefonte, PA, USA) for calibration, and stock 1000 mg/L single-element standard solutions of P (TraceCERT, Supelco, Bellefonte, PA, USA) were used for the preparation of working standard solutions of P for calibration.

The concentrations of 5 elements (As, B, Cd, Co, and Pb) in soil extracts, digested leaves, must, and wine samples were determined by inductively coupled plasma mass spectrometry (ICP-MS 7900, Agilent, Santa Clara, CA, USA) under optimal instrumental parameters [65]. Stock multielement standard solutions (ICP Multielement Standard Solution VI, CertiPUR, Supelco, Bellefonte, PA, USA) with initial concentrations of 10 mg/L for Cd, Cr, and Pb and 100 mg/L for As and B were used for the preparation of working standard solutions for calibration.

The concentrations of K, Li, and Na in soil extracts, digested leaves, must, and wine samples were determined by flame atomic-emission spectrometry (Perkin Elmer AAnalyst 400, Waltham, MA, USA) in air-acetylene flame using instrumental parameters recommended by the manufacturer. Single-element standard solutions of each of elements (Merck, Darmstadt, Germany) with initial concentrations of 1000 mg/L were used for calibration after appropriate dilution.

All standard solutions were prepared with Milli-Q water. For stabilization of the standard solutions, super-pure nitric acid was used.

The data presented in Tables S1–S4 were obtained as averages from analysis of three parallel samples and three replicates of each sample by the instrumental measurement. The RSD achieved was in the range 1–5%. The data are not presented as average ± standard deviation, because the uncertainty of these calculations comes mainly from the instrumental method used. The aim of this work is not to develop and validate a new analytical procedure. We used standard and well-known procedures for soil extractions, as well as leaf, must and wine acid mineralization, and we employed optimal instrumental parameters (FAES, ICP-OES, ICP-MS) recommended by the manufacturer. Soils and grapevines are complicated biosystems [22]. The uncertainties they exhibit (such as sampling and elements’ distribution) are many times more significant than the uncertainty due to instrumental method applied.

### 4.4. Statistical Analysis

The mean value is the calculated central value of a set of numbers and shows the tendency of the data.

The median is the value in the middle of the dataset, which means that 50% of the numbers are higher (or equal) to the median, and 50% of the values are smaller (or equal) to the median.

The standard deviations of the data sets were calculated. The standard deviation is a measure of the dispersion of the mean in the dataset.

Pearson coefficients (R) were calculated to assess the linear correlation relationships between sets of different elements in different sample matrixes. The correlation coefficient is a measure of the strength of the linear relationship between the data sets of two variables. The interval between −1 and 1 is the range of the values for the Pearson coefficient. If R is close to 1, it means there is a positive correlation; when R nears −1, it means that the two variables have a negative correlation; and when R is close to 0, it shows there is no significant correlation between the variables [66]. The null hypothesis of the Pearson correlation test is R = 0—there is no significant correlation; for 95% statistical significance the calculated probability (*p* value) is greater than 0.05. The alternative hypothesis is the following: If the correlation coefficient deviates significantly from 0 (*p* < 0.05), there is a linear correlation.

Method of least squares was used for curve fitting.

The decreasing percent (*D*%) of studied chemical elements were calculated as follows:

$$D\%=\frac{c\left(must\right)-c(wine)}{c(must)}\times 100,$$

where *c*(*must*) is the calculated element’s concentration in must; *c*(*wine*) is the calculated element’s concentration in wine.

All statistical calculations were performed by Microsoft Office Professional Plus 2019 (Excel).

The high-quality figures were constructed in the R environment [67] with the ggplot2 package v. 3.5.1. [68].

## 5. Conclusions

The values of calculated correlation coefficients between the concentrations of chemical elements in the system soil/grape/must/wine were used for the assessment of their consistency and relevance in tracing the geographical origin of wine. The elements exhibiting significant correlations between their bioavailable concentrations in soils and their final concentrations in wines such as Ba, K, Li, Pb, Sr, and Zn are strong candidates for geographical origin determination. Elements with weak or inconsistent correlations such as B, Ca, Cu, Fe, Mg, Na, P, Cd, Cr, and Ni were deemed less suitable descriptors. Their variability may be influenced by biological processes, winemaking practices, or environmental factors that obscure their relationship with soil composition. The set of optimal elements descriptors varied between red and white wines. The approach proposed by focusing on elements with clear and consistent behavior across the wine production process offers a more targeted and scientifically grounded method for identifying significant descriptors of wine origin. Further research and validation in diverse regions and wine types will enhance the reliability and applicability of this methodology.

## Figures and Tables

**Table 1:** Decreasing (*D*%) of macroelements’ content (minimal percent and maximal percent) in the system must/wine.

	Red Wines		White Wines	
Element	Min%	Max%	Min%	Max%
Al	4.92	94.71	7.72	86.65
B	2.68	33.34	9.77	34.24
Ba	23.36	96.01	10.99	94.45
Ca	4.96	67.49	16.58	59.61
Cu	54.45	99.62	7.60	97.65
Fe	11.35	84.27	17.10	64.92
K	5.76	59.16	28.59	67.04
Mg	9.47	34.38	8.81	44.36
Mn	8.28	60.54	15.76	73.53
Na	41.23	86.13	6.13	83.99
P	10.19	39.50	14.13	53.14
Sr	17.20	71.88	24.71	62.23
Zn	9.44	72.03	20.26	53.24

**Table 2:** Decreasing (*D*%) of microelements’ content (minimal percent and maximal percent) in the system must/wine.

	Red Wines		White Wines	
Element	Min%	Max%	Min%	Max%
As	56.52	94.32	10.35	93.03
Cd	22.22	94.92	20.00	75.00
Co	8.78	91.20	13.79	72.41
Cr	10.34	57.14	9.38	34.88
Li	14.29	64.52	24.00	45.45
Ni	44.25	90.08	32.91	90.67
Pb	23.08	44.44	23.08	44.44

**Table 3:** Characteristics of the samples.

Grapevine Variety	Sub-Regions *	Region	Sample Number
Chardonnay 1	Oryahovo (1)	Danubian plain	1
Chardonnay 2			2
Chardonnay 3			3
Chardonnay 4			4
Sauvignon Blanc			5
Viognier			6
Egiodola			7
Cabernet Franc			8
Cabernet Sauvignon			9
Marselan			10
Merlot			11
Pinot Noir 1			12
Pinot Noir 2			13
Syrah			14
Muscat Ottonel	Pirgovo (2)		15
Chardonnay	Suvorovo (3)	Black Sea Region	16
Sauvignon Blanc			17
Cabernet Sauvignon	Starosel (4)	Rose Valley	18
Chardonnay	Topoli dol (5)	Thracian Valley	19
Tamyanka			20
Cabernet Sauvignon			21
Syrah			22
Sauvignon Blanc	Brestnik (6)		23
Cabernet Franc			24
Syrah			25
Cabernet Sauvignon	Chernodab (7)		26
Cabernet Sauvignon	Levunovo (8)	Struma Valley	27
Merlot			28
Melnik	General Todorov (9)		29
Shiroka Melnishka Loza	Vranya (10)		30

* The numbers in brackets correspond to numbers on the map on Figure 3.

## Data Availability

The data presented in this study are available upon request from the corresponding author.

## Supplementary Materials

The following supporting information can be downloaded at https://www.mdpi.com/article/10.3390/molecules30030475/s1. Table S1: Macroelements’ content in red varieties. Table S2: Macroelements’ content in white varieties. Table S3: Microelements’ content in red varieties. Table S4: Microelements’ content in white varieties. Table S5: Significant correlation coefficients (*p* < 0.05).
