# Supplementary material for: From the Soil to the Wine—Elements’ Migration in Monovarietal Bulgarian Wines
Source: Molecules. 2025 Jan 22;30(3):475. doi: 10.3390/molecules30030475 (PMC11820015; doi:10.3390/molecules30030475)
Supplement: Supplementary file 1 [file molecules-30-00475-s001.zip › Table S1.pdf]

Table S1. Macroelements' content in red varieties.

Red varieties	Region	Element	Acetic [μg/g]	EDTA [μg/g]	Leaves [μg/g]	Must [mg/L]	Wine [mg/L]
Cabernet Franc	Oryahovo	Al	98	33	64	0.54	0.17
Cabernet Sauvignon	Oryahovo	Al	84	32	85	0.85	0.28
Egiodola	Oryahovo	Al	83	20	93	1.22	0.24
Marselan	Oryahovo	Al	76	31	94	0.67	0.26
Merlot	Oryahovo	Al	87	34	84	0.53	0.30
Pinot Noir	Oryahovo	Al	105	23	75	0.58	0.26
Pinot Noir	Oryahovo	Al	95	24	62	0.62	0.28
Syrah	Oryahovo	Al	79	29	74	0.78	0.19
Cabernet Sauvignon	Starosel	Al	281	213	62	0.41	0.11
Cabernet Sauvignon	Topoli dol	Al	124	72	68	0.33	0.30
Syrah	Topoli dol	Al	195	186	37	0.32	0.30
Cabernet Franc	Brestnik	Al	117	28	27	1.75	0.37
Syrah	Brestnik	Al	113	21	67	1.89	0.10
Cabernet Sauvignon	Chernodab	Al	150	124	72	0.31	0.29
Cabernet Sauvignon	Levunovo	Al	125	60	69	0.44	0.18
Merlot	Levunovo	Al	111	64	62	0.30	0.20
Melnik 55	General Todorov	Al	70	58	52	0.31	0.16
Broad-Leaved Melnik	Vranya	Al	117	77	62	1.22	0.39
		min	70	20	27	0.30	0.10
		max	281	213	94	1.89	0.39
		mean	117	63	67	0.73	0.24
		median	108	34	68	0.56	0.26
		st dev	51	57	17	0.49	0.08

Red varieties	Region	Element	Acetic [µg/g]	EDTA [µg/g]	Leaves [µg/g]	Must [mg/L]	Wine [mg/L]
Cabernet Franc	Oryahovo	B	1.69	0.48	33	7.2	6.0
Cabernet Sauvignon	Oryahovo	B	2.22	0.54	54	8.2	7.1
Egiodola	Oryahovo	B	2.21	0.68	25	7.7	6.5
Marselan	Oryahovo	B	2.84	0.86	44	8.1	6.6
Merlot	Oryahovo	B	1.24	0.42	62	9.3	9.1
Pinot Noir	Oryahovo	B	1.03	0.31	43	7.5	5.1
Pinot Noir	Oryahovo	B	1.84	0.50	67	6.8	5.5
Syrah	Oryahovo	B	2.41	0.72	68	6.5	5.4
Cabernet Sauvignon	Starosel	B	0.20	0.07	14	2.6	1.8
Cabernet Sauvignon	Topoli dol	B	0.10	0.11	23	5.8	4.8
Syrah	Topoli dol	B	0.20	0.09	24	7.0	6.3
Cabernet Franc	Brestnik	B	0.89	0.09	25	8.3	7.4
Syrah	Brestnik	B	1.01	0.08	26	6.1	5.1
Cabernet Sauvignon	Chernodab	B	0.20	0.07	26	8.5	7.0
Cabernet Sauvignon	Levunovo	B	0.20	0.54	20	6.1	5.6
Merlot	Levunovo	B	0.26	0.61	21	9.3	7.6
Melnik 55	General Todorov	B	0.10	0.09	29	9.4	7.5
Broad-Leaved Melnik	Vranya	B	0.60	0.10	28	4.3	2.9
		min	0.100	0.070	14	2.6	1.8
		max	2.8	0.86	68	9.4	9.1
		mean	1.07	0.35	35	7.1	6.0
		median	0.95	0.36	27	7.3	6.1
		st dev	0.92	0.27	17	1.8	1.7

Red varieties	Region	Element	Acetic [µg/g]	EDTA [µg/g]	Leaves [µg/g]	Must [mg/L]	Wine [mg/L]
Cabernet Franc	Oryahovo	Ba	21	17	9.1	0.076	0.045
Cabernet Sauvignon	Oryahovo	Ba	21	16	1.39	0.523	0.042
Egiodola	Oryahovo	Ba	21	4.8	9.5	0.122	0.039
Marselan	Oryahovo	Ba	23	22	9.1	0.447	0.059
Merlot	Oryahovo	Ba	20	22	7.1	0.067	0.043
Pinot Noir	Oryahovo	Ba	21	5.2	8.2	0.120	0.039
Pinot Noir	Oryahovo	Ba	23	16	9.0	0.093	0.037
Syrah	Oryahovo	Ba	21	18	11	0.067	0.051
Cabernet Sauvignon	Starosel	Ba	39	29	22	0.38	0.189
Cabernet Sauvignon	Topoli dol	Ba	82	107	27	0.38	0.240
Syrah	Topoli dol	Ba	71	120	61	0.46	0.049
Cabernet Franc	Brestnik	Ba	25	30	5.5	1.09	0.117
Syrah	Brestnik	Ba	34	23	9.1	1.31	0.052
Cabernet Sauvignon	Chernodab	Ba	42	82	29	0.39	0.248
Cabernet Sauvignon	Levunovo	Ba	40	44	34	0.121	0.020
Merlot	Levunovo	Ba	19	16	13	0.67	0.190
Melnik 55	General Todorov	Ba	32	46	12	0.139	0.020
Broad-Leaved Melnik	Vranya	Ba	40	48	15	0.40	0.048
		min	19.0	4.8	1.4	0.067	0.020
		max	81.6	120	61	1.31	0.25
		mean	33.0	37	16	0.38	0.085
		median	23.9	23	10	0.38	0.048
		st dev	17.8	33	14	0.35	0.077

Red varieties	Region	Element	Acetic [µg/g]	EDTA [µg/g]	Leaves [µg/g]	Must [mg/L]	Wine [mg/L]
Cabernet Franc	Oryahovo	Ca	61912	32096	28505	97	61
Cabernet Sauvignon	Oryahovo	Ca	56936	28696	29337	109	72
Egiodola	Oryahovo	Ca	87344	39858	26493	112	64
Marselan	Oryahovo	Ca	59952	35042	24794	119	61
Merlot	Oryahovo	Ca	45924	32460	22359	84	64
Pinot Noir	Oryahovo	Ca	71580	45486	21750	131	43
Pinot Noir	Oryahovo	Ca	57020	27846	23211	81	77
Syrah	Oryahovo	Ca	64304	34748	26376	73	56
Cabernet Sauvignon	Starosel	Ca	1299	723	14752	89	54
Cabernet Sauvignon	Topoli dol	Ca	5494	3724	20310	65	35
Syrah	Topoli dol	Ca	3700	4663	18931	69	53
Cabernet Franc	Brestnik	Ca	28764	20376	22414	108	42
Syrah	Brestnik	Ca	42316	20262	24432	151	59
Cabernet Sauvignon	Chernodab	Ca	3499	4362	19321	95	44
Cabernet Sauvignon	Levunovo	Ca	5302	4794	24536	74	40
Merlot	Levunovo	Ca	6397	4581	22847	81	50
Melnik 55	General Todorov	Ca	2052	2150	23256	87	49
Broad-Leaved Melnik	Vranya	Ca	8979	8896	22574	112	74
		min	1299	723	14752	65	35
		max	87344	45486	29337	151	77
		mean	34043	19487	23122	97	55
		median	35540	20319	23029	92	55
		st dev	29591	15241	3510	23	12

Red varieties	Region	Element	Acetic [µg/g]	EDTA [µg/g]	Leaves [µg/g]	Must [mg/L]	Wine [mg/L]
Cabernet Franc	Oryahovo	Cu	0.46	13	135	0.93	0.027
Cabernet Sauvignon	Oryahovo	Cu	0.41	14	216	0.63	0.015
Egiodola	Oryahovo	Cu	0.70	10	103	1.42	0.071
Marselan	Oryahovo	Cu	3.3	37	181	1.28	0.060
Merlot	Oryahovo	Cu	0.32	14	130	1.01	0.232
Pinot Noir	Oryahovo	Cu	0.82	8.8	159	0.56	0.126
Pinot Noir	Oryahovo	Cu	0.30	3.0	178	0.81	0.052
Syrah	Oryahovo	Cu	1.06	17	165	1.34	0.499
Cabernet Sauvignon	Starosel	Cu	3.7	7.0	2.0	0.36	0.001
Cabernet Sauvignon	Topoli dol	Cu	0.30	4.8	1.45	0.29	0.076
Syrah	Topoli dol	Cu	0.20	9.7	5.0	0.42	0.191
Cabernet Franc	Brestnik	Cu	7.6	46	84	0.42	0.042
Syrah	Brestnik	Cu	4.5	23	94	1.02	0.208
Cabernet Sauvignon	Chernodab	Cu	0.54	11	2.7	0.59	0.201
Cabernet Sauvignon	Levunovo	Cu	0.28	2.6	194	0.50	0.107
Merlot	Levunovo	Cu	0.20	3.8	114	0.68	0.262
Melnik 55	General Todorov	Cu	9.1	27	80	0.29	0.100
Broad-Leaved Melnik	Vranya	Cu	0.60	17	39	0.38	0.168
		min	0.20	2.6	1.45	0.29	0.001
		max	9.1	46	216	1.42	0.50
		mean	1.91	15	105	0.72	0.135
		median	0.57	12	108	0.61	0.104
		st dev	2.7	12	71	0.37	0.121

Red varieties	Region	Element	Acetic [µg/g]	EDTA [µg/g]	Leaves [µg/g]	Must [mg/L]	Wine [mg/L]
Cabernet Franc	Oryahovo	Fe	1.29	53	84	1.69	1.48
Cabernet Sauvignon	Oryahovo	Fe	1.15	52	149	0.93	0.56
Egiodola	Oryahovo	Fe	2.8	49	87	1.81	1.31
Marselan	Oryahovo	Fe	1.29	62	102	1.71	1.50
Merlot	Oryahovo	Fe	1.19	50	98	2.1	1.76
Pinot Noir	Oryahovo	Fe	1.96	40	88	1.90	1.30
Pinot Noir	Oryahovo	Fe	1.33	34	80	1.67	1.48
Syrah	Oryahovo	Fe	1.12	59	98	1.40	1.24
Cabernet Sauvignon	Starosel	Fe	9.9	151	61	2.2	1.67
Cabernet Sauvignon	Topoli dol	Fe	4.4	64	63	1.66	1.39
Syrah	Topoli dol	Fe	5.3	127	57	1.53	0.90
Cabernet Franc	Brestnik	Fe	1.68	42	46	1.57	1.12
Syrah	Brestnik	Fe	1.80	30	69	1.08	0.78
Cabernet Sauvignon	Chernodab	Fe	8.6	132	59	2.1	1.50
Cabernet Sauvignon	Levunovo	Fe	3.2	26	170	1.56	1.34
Merlot	Levunovo	Fe	2.3	30	94	0.87	0.54
Melnik 55	General Todorov	Fe	6.2	59	60	2.5	0.86
Broad-Leaved Melnik	Vranya	Fe	2.9	25	71	2.1	0.33
		min	1.1	25	46	0.87	0.33
		max	9.9	151	170	2.5	1.76
		mean	3.2	60	85	1.69	1.17
		median	2.1	51	82	1.68	1.30
		st dev	2.6	38	32	0.44	0.41

Red varieties	Region	Element	Acetic [µg/g]	EDTA [µg/g]	Leaves [µg/g]	Must [mg/L]	Wine [mg/L]
Cabernet Franc	Oryahovo	K	5.2	39	2050	1336	1014
Cabernet Sauvignon	Oryahovo	K	4.4	30	2125	949	894
Egiodola	Oryahovo	K	4.8	30	2875	1229	936
Marselan	Oryahovo	K	8.2	48	3300	1547	937
Merlot	Oryahovo	K	6.2	46	2250	1447	821
Pinot Noir	Oryahovo	K	3.5	20	3150	1679	1209
Pinot Noir	Oryahovo	K	2.7	6.1	2225	1129	809
Syrah	Oryahovo	K	6.1	51	2675	1632	970
Cabernet Sauvignon	Starosel	K	212	114	5775	1987	1138
Cabernet Sauvignon	Topoli dol	K	60	103	3425	1675	915
Syrah	Topoli dol	K	92	186	3200	1732	707
Cabernet Franc	Brestnik	K	137	175	5450	1558	1079
Syrah	Brestnik	K	137	192	6575	1835	1287
Cabernet Sauvignon	Chernodab	K	415	509	2625	2013	1532
Cabernet Sauvignon	Levunovo	K	44	44	3025	1974	1220
Merlot	Levunovo	K	69	65	5325	1622	894
Melnik 55	General Todorov	K	108	157	2625	1975	1011
Broad-Leaved Melnik	Vranya	K	100	124	4750	1654	739
		min	2.7	6.1	2050	949	707
		max	415	509	6575	2013	1532
		mean	79	108	3524	1610	1006
		median	52	58	3088	1643	953
		st dev	104	117	1406	304	209

Red varieties	Region	Element	Acetic [µg/g]	EDTA [µg/g]	Leaves [µg/g]	Must [mg/L]	Wine [mg/L]
Cabernet Franc	Oryahovo	Mg	10501	676	3715	84	69
Cabernet Sauvignon	Oryahovo	Mg	10480	680	4363	95	80
Egiodola	Oryahovo	Mg	12895	509	3882	97	78
Marselan	Oryahovo	Mg	12120	867	3903	102	90
Merlot	Oryahovo	Mg	13406	754	3450	99	77
Pinot Noir	Oryahovo	Mg	15948	616	3339	114	75
Pinot Noir	Oryahovo	Mg	9864	539	4077	86	71
Syrah	Oryahovo	Mg	11918	727	4403	104	89
Cabernet Sauvignon	Starosel	Mg	213	103	2124	88	71
Cabernet Sauvignon	Topoli dol	Mg	805	712	2737	104	76
Syrah	Topoli dol	Mg	1113	1285	2976	112	91
Cabernet Franc	Brestnik	Mg	411	225	1290	93	80
Syrah	Brestnik	Mg	519	193	1663	86	57
Cabernet Sauvignon	Chernodab	Mg	663	755	2375	91	72
Cabernet Sauvignon	Levunovo	Mg	1015	785	3238	91	79
Merlot	Levunovo	Mg	839	513	2867	102	82
Melnik 55	General Todorov	Mg	446	416	2422	85	77
Broad-Leaved Melnik	Vranya	Mg	507	318	1811	65	50
		min	213	103	1290	65	50
		max	15948	1285	4403	114	91
		mean	5759	593	3035	94	76
		median	1064	646	3107	94	77
		st dev	6013	282	944	12	10

Red varieties	Region	Element	Acetic [µg/g]	EDTA [µg/g]	Leaves [µg/g]	Must [mg/L]	Wine [mg/L]
Cabernet Franc	Oryahovo	Mn	122	54	68	1.10	0.88
Cabernet Sauvignon	Oryahovo	Mn	119	57	151	1.34	0.98
Egiodola	Oryahovo	Mn	145	48	64	1.23	0.84
Marselan	Oryahovo	Mn	140	82	82	1.45	1.01
Merlot	Oryahovo	Mn	121	63	77	1.65	1.04
Pinot Noir	Oryahovo	Mn	174	33	71	1.45	0.78
Pinot Noir	Oryahovo	Mn	176	22	87	1.45	0.87
Syrah	Oryahovo	Mn	136	69	102	1.67	1.01
Cabernet Sauvignon	Starosel	Mn	98	50	171	2.1	1.93
Cabernet Sauvignon	Topoli dol	Mn	75	125	69	2.1	1.67
Syrah	Topoli dol	Mn	90	125	116	1.90	1.51
Cabernet Franc	Brestnik	Mn	208	220	96	1.98	1.69
Syrah	Brestnik	Mn	168	74	165	2.7	1.06
Cabernet Sauvignon	Chernodab	Mn	136	360	153	1.87	1.40
Cabernet Sauvignon	Levunovo	Mn	51	62	89	1.77	1.29
Merlot	Levunovo	Mn	68	68	67	1.80	1.25
Melnik 55	General Todorov	Mn	101	144	98	1.93	1.57
Broad-Leaved Melnik	Vranya	Mn	96	132	44	0.56	0.39
		min	51	22	44	0.56	0.39
		max	208	360	171	2.7	1.93
		mean	123	99	98	1.67	1.18
		median	121	69	88	1.72	1.05
		st dev	41	81	38	0.47	0.39

Red varieties	Region	Element	Acetic [µg/g]	EDTA [µg/g]	Leaves [µg/g]	Must [mg/L]	Wine [mg/L]
Cabernet Franc	Oryahovo	Na	41	3.8	70	12	3.4
Cabernet Sauvignon	Oryahovo	Na	36	2.5	53	20	3.8
Egiodola	Oryahovo	Na	52	1.63	58	15	4.3
Marselan	Oryahovo	Na	44	4.0	52	21	3.5
Merlot	Oryahovo	Na	38	2.3	41	12	3.2
Pinot Noir	Oryahovo	Na	48	1.03	12	16	5.1
Pinot Noir	Oryahovo	Na	42	0.90	32	12	3.7
Syrah	Oryahovo	Na	39	3.1	41	13	3.7
Cabernet Sauvignon	Starosel	Na	10	2.2	103	18	8.9
Cabernet Sauvignon	Topoli dol	Na	23	0.91	59	12	3.9
Syrah	Topoli dol	Na	20	0.60	22	13	4.2
Cabernet Franc	Brestnik	Na	16	5.9	50	30	8.0
Syrah	Brestnik	Na	22	3.3	76	22	3.3
Cabernet Sauvignon	Chernodab	Na	13	1.12	54	14	7.9
Cabernet Sauvignon	Levunovo	Na	30	1.18	42	42	5.8
Merlot	Levunovo	Na	22	1.81	61	16	9.5
Melnik 55	General Todorov	Na	13	0.46	31	11	3.5
Broad-Leaved Melnik	Vranya	Na	20	0.89	37	10	3.1
		min	10	0.46	12	10	3.1
		max	52	5.9	103	42	9.5
		mean	29	2.1	50	17	4.9
		median	26	1.72	51	14	3.8
		st dev	13	1.46	21	7.9	2.1

Red varieties	Region	Element	Acetic [µg/g]	EDTA [µg/g]	Leaves [µg/g]	Must [mg/L]	Wine [mg/L]
Cabernet Franc	Oryahovo	P	80	76	1667	219	138
Cabernet Sauvignon	Oryahovo	P	46	50	1387	191	129
Egiodola	Oryahovo	P	29	33	1240	162	114
Marselan	Oryahovo	P	68	80	827	194	128
Merlot	Oryahovo	P	28	46	1120	187	156
Pinot Noir	Oryahovo	P	15	21	1360	131	118
Pinot Noir	Oryahovo	P	14	18	973	193	128
Syrah	Oryahovo	P	52	62	1573	245	148
Cabernet Sauvignon	Starosel	P	78	44	2040	229	152
Cabernet Sauvignon	Topoli dol	P	15	4.2	1707	231	144
Syrah	Topoli dol	P	22	8.1	1387	232	196
Cabernet Franc	Brestnik	P	85	119	1307	248	193
Syrah	Brestnik	P	92	65	2027	235	194
Cabernet Sauvignon	Chernodab	P	38	22	1356	253	186
Cabernet Sauvignon	Levunovo	P	76	32	827	160	114
Merlot	Levunovo	P	78	36	1093	132	91
Melnik 55	General Todorov	P	36	9.2	1257	222	159
Broad-Leaved Melnik	Vranya	P	71	18	1333	233	142
		min	14	4.2	827	131	91
		max	92	119	2040	253	196
		mean	51	41	1360	205	146
		median	49	34	1345	220	143
		st dev	27	30	347	39	30

Red varieties	Region	Element	Acetic [µg/g]	EDTA [µg/g]	Leaves [µg/g]	Must [mg/L]	Wine [mg/L]
Cabernet Franc	Oryahovo	Sr	53	22	133	0.66	0.37
Cabernet Sauvignon	Oryahovo	Sr	52	22	150	0.54	0.38
Egiodola	Oryahovo	Sr	65	21	176	0.47	0.39
Marselan	Oryahovo	Sr	47	30	136	0.52	0.31
Merlot	Oryahovo	Sr	60	21	118	0.65	0.42
Pinot Noir	Oryahovo	Sr	85	22	119	0.72	0.51
Pinot Noir	Oryahovo	Sr	68	12	136	0.69	0.39
Syrah	Oryahovo	Sr	47	26	133	0.37	0.21
Cabernet Sauvignon	Starosel	Sr	18	8.4	132	0.26	0.09
Cabernet Sauvignon	Topoli dol	Sr	18	18	239	0.42	0.18
Syrah	Topoli dol	Sr	28	35	304	0.35	0.22
Cabernet Franc	Brestnik	Sr	15	10	29	0.31	0.11
Syrah	Brestnik	Sr	19	8.7	32	0.32	0.09
Cabernet Sauvignon	Chernodab	Sr	9.2	11	181	0.21	0.09
Cabernet Sauvignon	Levunovo	Sr	18	16	275	0.33	0.19
Merlot	Levunovo	Sr	23	18	246	0.45	0.20
Melnik 55	General Todorov	Sr	12	13	40	0.19	0.09
Broad-Leaved Melnik	Vranya	Sr	11	10	55	0.18	0.07
		min	9.2	8.4	29	0.180	0.070
		max	85	35	304	0.72	0.51
		mean	36	18	146	0.42	0.24
		median	26	18	134	0.40	0.21
		st dev	24	7.6	81	0.175	0.141

Red varieties	Region	Element	Acetic [µg/g]	EDTA [µg/g]	Leaves [µg/g]	Must [mg/L]	Wine [mg/L]
Cabernet Franc	Oryahovo	Zn	1.13	3.0	16	0.89	0.67
Cabernet Sauvignon	Oryahovo	Zn	0.78	2.6	21	0.98	0.64
Egiodola	Oryahovo	Zn	0.80	2.6	12	0.86	0.48
Marselan	Oryahovo	Zn	1.78	3.9	19	1.23	0.84
Merlot	Oryahovo	Zn	0.63	3.2	21	0.57	0.39
Pinot Noir	Oryahovo	Zn	1.01	3.0	14	0.54	0.39
Pinot Noir	Oryahovo	Zn	0.14	1.8	5.8	0.61	0.41
Syrah	Oryahovo	Zn	0.95	3.1	9.2	0.81	0.60
Cabernet Sauvignon	Starosel	Zn	2.0	2.7	12	0.51	0.24
Cabernet Sauvignon	Topoli dol	Zn	0.61	2.5	10	0.59	0.31
Syrah	Topoli dol	Zn	0.63	3.3	13	0.64	0.44
Cabernet Franc	Brestnik	Zn	32	29	53	1.47	1.33
Syrah	Brestnik	Zn	26	18	39	1.19	0.33
Cabernet Sauvignon	Chernodab	Zn	2.3	6.4	12	0.38	0.18
Cabernet Sauvignon	Levunovo	Zn	0.57	2.1	18	0.34	0.16
Merlot	Levunovo	Zn	0.83	2.7	12	0.63	0.49
Melnik 55	General Todorov	Zn	12	6.6	7.2	1.41	1.04
Broad-Leaved Melnik	Vranya	Zn	11	2.9	11	1.47	1.21
		min	0.136	1.79	5.8	0.34	0.16
		max	32	29	53	1.5	1.3
		mean	5.3	5.5	17	0.84	0.56
		median	1.0	3.0	13	0.72	0.46
		st dev	9.4	7.0	12	0.37	0.34
