# Supplementary material for: From the Soil to the Wine—Elements’ Migration in Monovarietal Bulgarian Wines
Source: Molecules. 2025 Jan 22;30(3):475. doi: 10.3390/molecules30030475 (PMC11820015; doi:10.3390/molecules30030475)
Supplement: Supplementary file 1 [file molecules-30-00475-s001.zip › Table S2.pdf]

Table S2. Macroelements' content in white varieties.

White varieties	Region	Element	Acetic [µg/g]	EDTA [µg/g]	Leaves [µg/g]	Must [mg/L]	Wine [mg/L]
Chardonnay 1	Oryahovo	Al	92	36	75	0.50	0.23
Chardonnay 2	Oryahovo	Al	91	31	76	0.28	0.100
Chardonnay 3	Oryahovo	Al	84	28	62	0.53	0.31
Chardonnay 4	Oryahovo	Al	96	51	66	0.27	0.098
Sauvignon Blanc	Oryahovo	Al	87	28	71	0.22	0.130
Viognier	Oryahovo	Al	95	29	56	0.75	0.100
Muscat Ottonel	Pirgovo	Al	129	28	59	0.49	0.40
Chardonnay	Suvorovo	Al	136	91	80	0.71	0.49
Sauvignon Blanc	Suvorovo	Al	129	63	73	0.50	0.22
Chardonnay	Topoli dol	Al	182	164	44	0.16	0.148
Tamyanka	Topoli dol	Al	192	157	47	0.22	0.107
Sauvignon Blanc	Brestnik	Al	112	23	24	0.21	0.070
		min	84	23	24	0.160	0.070
		max	192	164	80	0.75	0.49
		mean	119	61	61	0.40	0.20
		median	104	34	64	0.39	0.14
		st dev	37	50	16	0.20	0.135

White varieties	Region	Element	Acetic [µg/g]	EDTA [µg/g]	Leaves [µg/g]	Must [mg/L]	Wine [mg/L]
Chardonnay 1	Oryahovo	B	1.36	0.39	51	6.6	5.3
Chardonnay 2	Oryahovo	B	1.31	0.45	53	5.6	3.9
Chardonnay 3	Oryahovo	B	1.74	0.38	58	7.8	6.0
Chardonnay 4	Oryahovo	B	2.4	0.51	47	7.3	5.3
Sauvignon Blanc	Oryahovo	B	1.70	0.44	50	7.6	5.6
Viognier	Oryahovo	B	2.5	0.94	54	6.8	5.8
Muscat Ottonel	Pirgovo	B	1.14	1.76	48	4.3	3.9
Chardonnay	Suvorovo	B	4.6	1.68	49	7.8	6.3
Sauvignon Blanc	Suvorovo	B	4.3	1.53	39	5.9	4.2
Chardonnay	Topoli dol	B	0.33	0.15	44	5.8	3.8
Tamyanka	Topoli dol	B	0.20	0.12	21	6.9	5.8
Sauvignon Blanc	Brestnik	B	0.90	0.10	22	5.7	4.8
		min	0.20	0.100	21	4.3	3.8
		max	4.6	1.76	58	7.8	6.3
		mean	1.87	0.71	45	6.5	5.1
		median	1.53	0.45	49	6.7	5.3
		st dev	1.38	0.62	12	1.07	0.90

White varieties	Region	Element	Acetic [μg/g]	EDTA [μg/g]	Leaves [μg/g]	Must [mg/L]	Wine [mg/L]
Chardonnay 1	Oryahovo	Ba	20	23	9.7	0.20	0.017
Chardonnay 2	Oryahovo	Ba	20	18	8.4	0.094	0.012
Chardonnay 3	Oryahovo	Ba	20	17	8.9	0.114	0.020
Chardonnay 4	Oryahovo	Ba	26	22	10	0.066	0.017
Sauvignon Blanc	Oryahovo	Ba	20	16	8.8	0.048	0.030
Viognier	Oryahovo	Ba	22	15	6.2	0.20	0.180
Muscat Ottonel	Pirgovo	Ba	31	8.9	15	0.108	0.032
Chardonnay	Suvorovo	Ba	41	63	13	0.082	0.040
Sauvignon Blanc	Suvorovo	Ba	37	42	13	0.51	0.028
Chardonnay	Topoli dol	Ba	75	101	26	0.25	0.135
Tamyanka	Topoli dol	Ba	78	137	72	0.31	0.162
Sauvignon Blanc	Brestnik	Ba	25	18	7.0	0.26	0.180
		min	20	8.9	6.2	0.048	0.012
		max	78	137	72	0.51	0.180
		mean	35	40	16	0.187	0.071
		median	25	20	10	0.156	0.031
		st dev	21	40	18	0.133	0.070

White varieties	Region	Element	Acetic [μg/g]	EDTA [μg/g]	Leaves [μg/g]	Must [mg/L]	Wine [mg/L]
Chardonnay 1	Oryahovo	Ca	40180	27286	27169	88	48
Chardonnay 2	Oryahovo	Ca	62224	37650	25327	90	58
Chardonnay 3	Oryahovo	Ca	51228	25276	27033	116	76
Chardonnay 4	Oryahovo	Ca	49201	26646	29345	107	69
Sauvignon Blanc	Oryahovo	Ca	54204	26084	27886	89	65
Viognier	Oryahovo	Ca	79776	39330	18294	109	44
Muscat Ottonel	Pirgovo	Ca	69832	43292	25510	112	68
Chardonnay	Suvorovo	Ca	26950	27034	25293	83	56
Sauvignon Blanc	Suvorovo	Ca	51092	38880	30519	73	40
Chardonnay	Topoli dol	Ca	4367	3812	23559	114	56
Tamyanka	Topoli dol	Ca	4205	5408	25938	62	52
Sauvignon Blanc	Brestnik	Ca	34458	15927	24956	105	51
		min	4205	3812	18294	62	40
		max	79776	43292	30519	116	76
		mean	43976	26385	25902	96	57
		median	50147	26840	25724	97	56
		st dev	23479	12785	3091	17	11

White varieties	Region	Element	Acetic [µg/g]	EDTA [µg/g]	Leaves [µg/g]	Must [mg/L]	Wine [mg/L]
Chardonnay 1	Oryahovo	Cu	2.0	29	147	1.13	0.05
Chardonnay 2	Oryahovo	Cu	1.06	17	196	1.21	0.03
Chardonnay 3	Oryahovo	Cu	9.6	48	194	2.1	0.11
Chardonnay 4	Oryahovo	Cu	8.2	94	173	0.46	0.07
Sauvignon Blanc	Oryahovo	Cu	1.79	20	192	0.43	0.16
Viognier	Oryahovo	Cu	2.9	30	139	0.59	0.11
Muscat Ottonel	Pirgovo	Cu	1.14	15	4.6	0.13	0.03
Chardonnay	Suvorovo	Cu	0.23	12	87	0.59	0.13
Sauvignon Blanc	Suvorovo	Cu	0.20	17	96	0.29	0.08
Chardonnay	Topoli dol	Cu	0.30	7.1	2.3	0.51	0.01
Tamyanka	Topoli dol	Cu	0.21	7.2	3.8	0.23	0.04
Sauvignon Blanc	Brestnik	Cu	8.1	23	97	0.21	0.19
		min	0.20	7.1	2.3	0.132	0.015
		max	9.6	94	196	2.1	0.194
		mean	3.0	27	111	0.65	0.084
		median	1.5	19	118	0.48	0.077
		st dev	3.5	24	75	0.56	0.056

White varieties	Region	Element	Acetic [µg/g]	EDTA [µg/g]	Leaves [µg/g]	Must [mg/L]	Wine [mg/L]
Chardonnay 1	Oryahovo	Fe	1.46	57	81	0.47	0.22
Chardonnay 2	Oryahovo	Fe	2.1	63	71	0.35	0.16
Chardonnay 3	Oryahovo	Fe	2.1	48	84	1.36	0.76
Chardonnay 4	Oryahovo	Fe	3.0	68	65	0.64	0.47
Sauvignon Blanc	Oryahovo	Fe	1.54	44	80	0.64	0.48
Viognier	Oryahovo	Fe	2.7	71	74	0.86	0.30
Muscat Ottonel	Pirgovo	Fe	1.83	28	81	0.82	0.68
Chardonnay	Suvorovo	Fe	0.98	79	80	0.67	0.50
Sauvignon Blanc	Suvorovo	Fe	1.38	92	87	1.15	0.63
Chardonnay	Topoli dol	Fe	3.4	106	50	0.91	0.56
Tamyanka	Topoli dol	Fe	4.6	99	66	0.87	0.55
Sauvignon Blanc	Brestnik	Fe	1.24	33	46	1.57	1.30
		min	0.98	28	46	0.35	0.16
		max	4.6	106	87	1.57	1.30
		mean	2.2	66	72	0.86	0.55
		median	1.94	66	77	0.84	0.53
		st dev	1.06	25	13	0.35	0.30

White varieties	Region	Element	Acetic [µg/g]	EDTA [µg/g]	Leaves [µg/g]	Must [mg/L]	Wine [mg/L]
Chardonnay 1	Oryahovo	K	7.1	54	3350	1603	685
Chardonnay 2	Oryahovo	K	6.5	45	3225	1716	857
Chardonnay 3	Oryahovo	K	2.7	16	2800	2014	895
Chardonnay 4	Oryahovo	K	7.1	49	4325	1399	999
Sauvignon Blanc	Oryahovo	K	3.2	19	3800	1113	699
Viognier	Oryahovo	K	5.1	35	3125	1392	773
Muscat Ottonel	Pirgovo	K	8.0	63	3825	1268	561
Chardonnay	Suvorovo	K	45	179	3125	1236	663
Sauvignon Blanc	Suvorovo	K	56	185	3325	1812	597
Chardonnay	Topoli dol	K	73	160	2725	1356	850
Tamyanka	Topoli dol	K	63	139	3225	1432	608
Sauvignon Blanc	Brestnik	K	125	157	6643	1643	692
		min	2.7	16	2725	1113	561
		max	125	185	6643	2014	999
		mean	33	92	3624	1499	740
		median	7.6	58	3275	1415	695
		st dev	39	66	1051	263	135

White varieties	Region	Element	Acetic [µg/g]	EDTA [µg/g]	Leaves [µg/g]	Must [mg/L]	Wine [mg/L]
Chardonnay 1	Oryahovo	Mg	7129	763	3817	82	70
Chardonnay 2	Oryahovo	Mg	9761	674	2856	62	46
Chardonnay 3	Oryahovo	Mg	8342	589	3515	97	68
Chardonnay 4	Oryahovo	Mg	6923	865	2875	78	63
Sauvignon Blanc	Oryahovo	Mg	10292	624	3117	97	57
Viognier	Oryahovo	Mg	15650	706	3717	107	60
Muscat Ottonel	Pirgovo	Mg	5215	631	3764	64	50
Chardonnay	Suvorovo	Mg	1175	485	3328	87	79
Sauvignon Blanc	Suvorovo	Mg	1528	622	2961	104	86
Chardonnay	Topoli dol	Mg	552	414	3203	94	80
Tamyanka	Topoli dol	Mg	1004	1157	2708	86	60
Sauvignon Blanc	Brestnik	Mg	373	124	1477	85	77
		min	373	124	1477	62	46
		max	15650	1157	3817	107	86
		mean	5662	638	3111	87	66
		median	6069	627	3160	87	65
		st dev	4882	249	639	14	13

White varieties	Region	Element	Acetic [µg/g]	EDTA [µg/g]	Leaves [µg/g]	Must [mg/L]	Wine [mg/L]
Chardonnay 1	Oryahovo	Mn	121	81	84	1.11	0.50
Chardonnay 2	Oryahovo	Mn	134	64	84	1.29	0.41
Chardonnay 3	Oryahovo	Mn	128	59	92	1.74	0.69
Chardonnay 4	Oryahovo	Mn	247	148	73	1.54	0.57
Sauvignon Blanc	Oryahovo	Mn	127	54	88	1.00	0.48
Viognier	Oryahovo	Mn	182	78	64	1.96	0.52
Muscat Ottonel	Pirgovo	Mn	82	30	176	1.87	1.44
Chardonnay	Suvorovo	Mn	108	216	312	2.3	1.91
Sauvignon Blanc	Suvorovo	Mn	141	121	214	1.47	1.04
Chardonnay	Topoli dol	Mn	103	120	100	0.93	0.54
Tamyanka	Topoli dol	Mn	88	121	163	1.68	0.99
Sauvignon Blanc	Brestnik	Mn	177	77	115	2.2	1.85
		min	82	30	64	0.93	0.41
		max	247	216	312	2.29	1.91
		mean	137	98	130	1.59	0.91
		median	127	79	96	1.61	0.63
		st dev	46	51	74	0.45	0.54

White varieties	Region	Element	Acetic [µg/g]	EDTA [µg/g]	Leaves [µg/g]	Must [mg/L]	Wine [mg/L]
Chardonnay 1	Oryahovo	Na	29	3.4	59	15	2.8
Chardonnay 2	Oryahovo	Na	33	3.2	31	18	3.1
Chardonnay 3	Oryahovo	Na	30	2.7	23	19	3.7
Chardonnay 4	Oryahovo	Na	27	3.6	24	10	2.7
Sauvignon Blanc	Oryahovo	Na	37	2.3	55	15	4.4
Viognier	Oryahovo	Na	44	3.5	28	15	2.4
Muscat Ottonel	Pirgovo	Na	41	1.31	45	15	14
Chardonnay	Suvorovo	Na	29	3.7	95	15	5.8
Sauvignon Blanc	Suvorovo	Na	38	5.1	114	21	4.5
Chardonnay	Topoli dol	Na	20	1.23	21	12	8.2
Tamyanka	Topoli dol	Na	28	0.98	24	11	3.1
Sauvignon Blanc	Brestnik	Na	21	3.5	12	19	8.9
		min	20	1.0	12	10	2.4
		max	44	5.1	114	21	14
		mean	31	2.9	44	16	5.3
		median	30	3.3	30	15	4.0
		st dev	7.4	1.2	32	3.3	3.4

White varieties	Region	Element	Acetic [μg/g]	EDTA [μg/g]	Leaves [μg/g]	Must [mg/L]	Wine [mg/L]
Chardonnay 1	Oryahovo	P	50	69	1333	184	140
Chardonnay 2	Oryahovo	P	51	65	1333	166	92
Chardonnay 3	Oryahovo	P	51	55	1480	210	159
Chardonnay 4	Oryahovo	P	214	193	1400	163	140
Sauvignon Blanc	Oryahovo	P	39	45	1627	124	93
Viognier	Oryahovo	P	159	150	1653	217	165
Muscat Ottonel	Pirgovo	P	38	26	1307	116	82
Chardonnay	Suvorovo	P	79	73	1600	191	132
Sauvignon Blanc	Suvorovo	P	94	101	1400	94	52
Chardonnay	Topoli dol	P	19	25	1347	233	184
Tamyanka	Topoli dol	P	21	8	1107	131	61
Sauvignon Blanc	Brestnik	P	64	51	1144	242	190
		min	19	7.6	1107	94	52
		max	214	193	1653	242	190
		mean	73	72	1394	173	124
		median	51	60	1373	175	136
		st dev	58	53	174	49	47

White varieties	Region	Element	Acetic [μg/g]	EDTA [μg/g]	Leaves [μg/g]	Must [mg/L]	Wine [mg/L]
Chardonnay 1	Oryahovo	Sr	61	22	154	0.58	0.43
Chardonnay 2	Oryahovo	Sr	47	28	87	0.41	0.23
Chardonnay 3	Oryahovo	Sr	69	19	122	0.55	0.41
Chardonnay 4	Oryahovo	Sr	55	16	135	0.44	0.32
Sauvignon Blanc	Oryahovo	Sr	40	19	114	0.49	0.19
Viognier	Oryahovo	Sr	64	32	137	0.65	0.38
Muscat Ottonel	Pirgovo	Sr	45	20	248	0.53	0.33
Chardonnay	Suvorovo	Sr	88	36	381	0.74	0.52
Sauvignon Blanc	Suvorovo	Sr	68	48	224	0.56	0.42
Chardonnay	Topoli dol	Sr	20	19	41	0.26	0.12
Tamyanka	Topoli dol	Sr	23	29	218	0.52	0.21
Sauvignon Blanc	Brestnik	Sr	16	6.6	35	0.28	0.12
		min	16	6.6	35	0.26	0.120
		max	88	48	381	0.74	0.52
		mean	50	25	158	0.50	0.31
		median	51	21	136	0.52	0.32
		st dev	22	11	97	0.139	0.131

White varieties	Region	Element	Acetic [µg/g]	EDTA [µg/g]	Leaves [µg/g]	Must [mg/L]	Wine [mg/L]
Chardonnay 1	Oryahovo	Zn	1.34	3.8	19	0.70	0.52
Chardonnay 2	Oryahovo	Zn	1.60	3.6	12	0.51	0.24
Chardonnay 3	Oryahovo	Zn	2.5	3.4	5.9	0.77	0.46
Chardonnay 4	Oryahovo	Zn	5.3	7.6	11	0.84	0.39
Sauvignon Blanc	Oryahovo	Zn	1.13	2.6	14	0.74	0.56
Viognier	Oryahovo	Zn	1.6	3.8	10	1.12	0.57
Muscat Ottonel	Pirgovo	Zn	3.4	3.5	16	0.80	0.47
Chardonnay	Suvorovo	Zn	0.18	3.6	16	1.10	0.73
Sauvignon Blanc	Suvorovo	Zn	0.80	4.3	15	0.63	0.45
Chardonnay	Topoli dol	Zn	0.30	3.3	12	0.95	0.76
Tamyanka	Topoli dol	Zn	0.49	3.0	13	0.78	0.37
Sauvignon Blanc	Brestnik	Zn	27	14	26	0.51	0.35
		min	0.18	2.6	5.9	0.51	0.24
		max	27	14	26	1.12	0.76
		mean	3.8	4.7	14	0.79	0.49
		median	1.47	3.6	14	0.77	0.46
		st dev	7.4	3.2	5.1	0.20	0.15
