# Supplementary material for: From the Soil to the Wine—Elements’ Migration in Monovarietal Bulgarian Wines
Source: Molecules. 2025 Jan 22;30(3):475. doi: 10.3390/molecules30030475 (PMC11820015; doi:10.3390/molecules30030475)
Supplement: Supplementary file 1 [file molecules-30-00475-s001.zip › Table S3.pdf]

Table S3. Microelements' content in red varieties.

Red varieties	Region	Element	Acetic [µg/g]	EDTA [µg/g]	Leaves [µg/g]	Must [mg/L]	Wine [mg/L]
Cabernet Franc	Oryahovo	As	4.0	5.0	NA	4.7	0.50
Cabernet Sauvignon	Oryahovo	As	5.0	7.0	NA	5.0	0.50
Egiodola	Oryahovo	As	3.0	6.0	NA	5.2	0.50
Marselan	Oryahovo	As	5.0	8.0	NA	4.7	0.75
Merlot	Oryahovo	As	3.0	6.0	NA	4.9	0.60
Pinot Noir	Oryahovo	As	4.0	7.0	NA	7.4	0.42
Pinot Noir	Oryahovo	As	4.0	6.0	NA	4.7	0.53
Syrah	Oryahovo	As	4.0	7.0	NA	4.6	0.60
Cabernet Sauvignon	Starosel	As	5.0	8.0	NA	1.23	0.50
Cabernet Sauvignon	Topoli dol	As	3.0	6.0	NA	5.2	1.00
Syrah	Topoli dol	As	5.0	7.0	NA	2.5	1.10
Cabernet Franc	Brestnik	As	5.0	6.0	NA	4.6	2.00
Syrah	Brestnik	As	4.0	6.0	NA	5.6	1.20
Cabernet Sauvignon	Chernodab	As	4.0	6.0	NA	6.1	0.70
Cabernet Sauvignon	Levunovo	As	11	15	NA	4.4	0.60
Merlot	Levunovo	As	9	13	NA	6.2	0.80
Melnik 55	General Todorov	As	5.0	7.0	NA	1.89	0.70
Broad-Leaved Melnik	Vranya	As	5.0	8.0	NA	1.55	0.50
		min	3.0	5.0	NA	1.23	0.42
		max	11	15	NA	7.4	2.0
		mean	4.9	7.4	NA	4.5	0.75
		median	4.5	7.0	NA	4.7	0.60
		st dev	2.0	2.5	NA	1.66	0.38

Red varieties	Region	Element	Acetic [µg/g]	EDTA [µg/g]	Leaves [µg/g]	Must [mg/L]	Wine [mg/L]
Cabernet Franc	Oryahovo	Cd	15	16	0.060	0.110	0.070
Cabernet Sauvignon	Oryahovo	Cd	11	17	0.080	0.120	0.070
Egiodola	Oryahovo	Cd	9.0	18	0.080	0.150	0.080
Marselan	Oryahovo	Cd	9.0	18	0.050	0.130	0.090
Merlot	Oryahovo	Cd	12	19	0.030	0.120	0.080
Pinot Noir	Oryahovo	Cd	11	21	0.020	0.110	0.070
Pinot Noir	Oryahovo	Cd	16	20	0.040	0.090	0.070
Syrah	Oryahovo	Cd	14	16	0.090	0.130	0.080
Cabernet Sauvignon	Starosel	Cd	7.0	14	0.030	0.43	0.120
Cabernet Sauvignon	Topoli dol	Cd	7.0	11	0.080	0.63	0.20
Syrah	Topoli dol	Cd	9.0	13	0.070	0.59	0.030
Cabernet Franc	Brestnik	Cd	12	12	0.030	3.7	2.2
Syrah	Brestnik	Cd	12	11	0.080	3.5	1.6
Cabernet Sauvignon	Chernodab	Cd	9.0	15	0.030	0.67	0.090
Cabernet Sauvignon	Levunovo	Cd	7.0	12	0.040	0.090	0.030
Merlot	Levunovo	Cd	6.0	15	0.030	0.140	0.070
Melnik 55	General Todorov	Cd	9.0	16	0.020	1.35	0.42
Broad-Leaved Melnik	Vranya	Cd	9.0	12	0.090	1.28	0.30
		min	6.0	11	0.020	0.090	0.030
		max	16	21	0.090	3.7	2.2
		mean	10	15	0.053	0.74	0.32
		median	9.0	16	0.045	0.145	0.080
		st dev	2.9	3.1	0.026	1.11	0.59

Red varieties	Region	Element	Acetic [µg/g]	EDTA [µg/g]	Leaves [µg/g]	Must [mg/L]	Wine [mg/L]
Cabernet Franc	Oryahovo	Co	0.012	0.021	NA	3.3	0.60
Cabernet Sauvignon	Oryahovo	Co	0.022	0.106	NA	2.6	0.80
Egiodola	Oryahovo	Co	0.35	0.140	NA	3.1	0.70
Marselan	Oryahovo	Co	0.034	0.045	NA	3.4	0.90
Merlot	Oryahovo	Co	0.110	0.24	NA	3.8	0.90
Pinot Noir	Oryahovo	Co	0.39	0.056	NA	3.1	0.60
Pinot Noir	Oryahovo	Co	0.022	0.045	NA	3.2	0.70
Syrah	Oryahovo	Co	0.089	0.096	NA	2.1	0.80
Cabernet Sauvignon	Starosel	Co	1.33	0.58	NA	2.3	0.20
Cabernet Sauvignon	Topoli dol	Co	0.056	2.3	NA	2.1	0.40
Syrah	Topoli dol	Co	0.81	1.88	NA	2.5	2.3
Cabernet Franc	Brestnik	Co	0.016	0.71	NA	1.87	0.60
Syrah	Brestnik	Co	0.30	0.52	NA	1.80	0.40
Cabernet Sauvignon	Chernodab	Co	0.85	4.0	NA	6.6	0.77
Cabernet Sauvignon	Levunovo	Co	0.020	0.82	NA	1.80	0.30
Merlot	Levunovo	Co	0.032	0.37	NA	1.70	0.80
Melnik 55	General Todorov	Co	0.91	3.4	NA	1.23	0.90
Broad-Leaved Melnik	Vranya	Co	0.23	1.51	NA	2.9	0.90
		min	0.012	0.021	NA	1.23	0.20
		max	1.328	4.0	NA	6.6	2.3
		mean	0.310	0.94	NA	2.7	0.75
		median	0.100	0.45	NA	2.6	0.74
		st dev	0.397	1.21	NA	1.20	0.44

Red varieties	Region	Element	Acetic [µg/g]	EDTA [µg/g]	Leaves [µg/g]	Must [mg/L]	Wine [mg/L]
Cabernet Franc	Oryahovo	Cr	2.1	0.58	NA	77	35
Cabernet Sauvignon	Oryahovo	Cr	2.1	0.16	NA	65	43
Egiodola	Oryahovo	Cr	2.0	0.39	NA	57	35
Marselan	Oryahovo	Cr	1.36	0.94	NA	52	37
Merlot	Oryahovo	Cr	0.30	0.10	NA	83	61
Pinot Noir	Oryahovo	Cr	4.5	0.17	NA	32	23
Pinot Noir	Oryahovo	Cr	2.1	0.25	NA	44	36
Syrah	Oryahovo	Cr	0.28	0.35	NA	52	36
Cabernet Sauvignon	Starosel	Cr	2.1	0.20	NA	29	26
Cabernet Sauvignon	Topoli dol	Cr	0.26	0.17	NA	44	32
Syrah	Topoli dol	Cr	1.57	0.10	NA	53	45
Cabernet Franc	Brestnik	Cr	0.96	0.54	NA	35	28
Syrah	Brestnik	Cr	0.87	1.94	NA	56	24
Cabernet Sauvignon	Chernodab	Cr	1.44	0.68	NA	58	28
Cabernet Sauvignon	Levunovo	Cr	0.10	0.54	NA	47	35
Merlot	Levunovo	Cr	0.24	0.74	NA	49	38
Melnik 55	General Todorov	Cr	0.80	0.65	NA	54	44
Broad-Leaved Melnik	Vranya	Cr	0.10	0.26	NA	36	27
		min	0.10	0.10	NA	29	23
		max	4.49	1.94	NA	83	61
		mean	1.29	0.49	NA	51	35
		median	1.16	0.37	NA	52	35
		st dev	1.12	0.44	NA	14	9.2

Red varieties	Region	Element	Acetic [µg/g]	EDTA [µg/g]	Leaves [µg/g]	Must [µg/L]	Wine [µg/L]
Cabernet Franc	Oryahovo	Li	8.1	2.8	39	19	13
Cabernet Sauvignon	Oryahovo	Li	7.6	2.6	42	18	13
Egiodola	Oryahovo	Li	12	3.5	30	21	18
Marselan	Oryahovo	Li	8.4	3.1	34	17	12
Merlot	Oryahovo	Li	6.1	2.8	29	15	11
Pinot Noir	Oryahovo	Li	12	3.9	27	19	16
Pinot Noir	Oryahovo	Li	6.7	2.3	40	17	12
Syrah	Oryahovo	Li	8.8	3.2	45	21	14
Cabernet Sauvignon	Starosel	Li	0.080	0.140	25	2.3	1.0
Cabernet Sauvignon	Topoli dol	Li	0.92	0.72	21	2.9	1.9
Syrah	Topoli dol	Li	0.72	0.82	31	2.4	1.8
Cabernet Franc	Brestnik	Li	2.3	1.78	24	3.2	2.1
Syrah	Brestnik	Li	3.3	1.68	27	3.1	1.9
Cabernet Sauvignon	Chernodab	Li	0.40	0.60	33	2.9	1.8
Cabernet Sauvignon	Levunovo	Li	0.84	0.62	38	4.3	2.1
Merlot	Levunovo	Li	1.60	1.20	30	3.8	2.0
Melnik 55	General Todorov	Li	2.3	0.78	21	3.1	1.1
Broad-Leaved Melnik	Vranya	Li	0.16	0.34	23	2.8	1.3
		min	0.08	0.140	21	2.3	1.0
		max	12.4	3.9	45	21	18
		mean	4.59	1.82	31	9.9	7.0
		median	2.80	1.73	30	4.1	2.1
		st dev	4.2	1.21	7.3	7.9	6.3

Red varieties	Region	Element	Acetic [µg/g]	EDTA [µg/g]	Leaves [µg/g]	Must [µg/L]	Wine [µg/L]
Cabernet Franc	Oryahovo	Ni	1.88	1.59	NA	61	6.0
Cabernet Sauvignon	Oryahovo	Ni	2.3	1.16	NA	51	7.9
Egiodola	Oryahovo	Ni	2.2	1.81	NA	40	7.0
Marselan	Oryahovo	Ni	2.7	1.90	NA	50	8.0
Merlot	Oryahovo	Ni	2.3	2.0	NA	69	8.4
Pinot Noir	Oryahovo	Ni	2.1	0.71	NA	45	4.5
Pinot Noir	Oryahovo	Ni	0.11	0.55	NA	51	7.3
Syrah	Oryahovo	Ni	1.82	1.85	NA	46	5.9
Cabernet Sauvignon	Starosel	Ni	0.12	1.09	NA	22	9.6
Cabernet Sauvignon	Topoli dol	Ni	1.8	1.44	NA	22	7.5
Syrah	Topoli dol	Ni	1.8	2.3	NA	25	14
Cabernet Franc	Brestnik	Ni	8.2	4.9	NA	18	7.8
Syrah	Brestnik	Ni	6.7	2.1	NA	54	6.3
Cabernet Sauvignon	Chernodab	Ni	1.6	4.4	NA	42	12
Cabernet Sauvignon	Levunovo	Ni	1.3	0.96	NA	24	12
Merlot	Levunovo	Ni	1.9	1.67	NA	22	11
Melnik 55	General Todorov	Ni	2.6	3.8	NA	36	18
Broad-Leaved Melnik	Vranya	Ni	0.14	1.79	NA	21	5.7
		min	0.11	0.55	NA	18	4.5
		max	8.2	4.9	NA	69	18
		mean	2.3	2.0	NA	39	8.8
		median	1.91	1.80	NA	41	7.9
		st dev	2.0	1.21	NA	16	3.4

Red varieties	Region	Element	Acetic [µg/g]	EDTA [µg/g]	Leaves [µg/g]	Must [µg/L]	Wine [µg/L]
Cabernet Franc	Oryahovo	Pb	0.13	2.5	0.13	8.0	5.0
Cabernet Sauvignon	Oryahovo	Pb	0.01	1.81	0.11	11	7.0
Egiodola	Oryahovo	Pb	0.13	0.70	0.22	47	36
Marselan	Oryahovo	Pb	0.21	3.2	0.16	22	12
Merlot	Oryahovo	Pb	0.24	0.78	0.23	80	50
Pinot Noir	Oryahovo	Pb	0.19	0.30	0.22	37	23
Pinot Noir	Oryahovo	Pb	0.08	0.23	0.32	13	8.6
Syrah	Oryahovo	Pb	0.12	1.79	0.21	29	17
Cabernet Sauvignon	Starosel	Pb	0.45	3.0	0.09	158	99
Cabernet Sauvignon	Topoli dol	Pb	0.14	3.2	0.11	6.6	4.4
Syrah	Topoli dol	Pb	0.26	2.7	0.21	7.7	5.1
Cabernet Franc	Brestnik	Pb	0.91	41	2.8	338	225
Syrah	Brestnik	Pb	1.14	26	1.9	322	179
Cabernet Sauvignon	Chernodab	Pb	0.65	4.9	0.11	4.8	3.0
Cabernet Sauvignon	Levunovo	Pb	0.23	1.42	0.09	13	8.0
Merlot	Levunovo	Pb	0.18	1.91	0.08	13	8.0
Melnik 55	General Todorov	Pb	0.58	3.1	0.13	11	7.0
Broad-Leaved Melnik	Vranya	Pb	0.90	3.3	0.22	104	69
		min	0.01	0.23	0.08	4.8	3.0
		max	1.14	41	2.80	338	225
		mean	0.36	5.7	0.41	68	43
		median	0.22	2.6	0.19	17	10
		st dev	0.33	11	0.73	104	64
