# Supplementary material for: From the Soil to the Wine—Elements’ Migration in Monovarietal Bulgarian Wines
Source: Molecules. 2025 Jan 22;30(3):475. doi: 10.3390/molecules30030475 (PMC11820015; doi:10.3390/molecules30030475)
Supplement: Supplementary file 1 [file molecules-30-00475-s001.zip › Table S4.pdf]

Table S4. Microelements' content in white varieties.

White varieties	Region	Element	Acetic [µg/g]	EDTA [µg/g]	Leaves [µg/g]	Must [µg/L]	Wine [µg/L]
Chardonnay 1	Oryahovo	As	5.0	8.0	NA	4.3	0.50
Chardonnay 2	Oryahovo	As	3.0	7.0	NA	5.2	0.60
Chardonnay 3	Oryahovo	As	5.0	6.0	NA	4.8	0.50
Chardonnay 4	Oryahovo	As	3.0	8.0	NA	4.4	0.50
Sauvignon Blanc	Oryahovo	As	3.0	8.0	NA	5.2	0.70
Viognier	Oryahovo	As	6.0	7.0	NA	7.2	0.50
Muscat Ottonel	Pirgovo	As	4.0	8.0	NA	4.6	1.70
Chardonnay	Suvorovo	As	8.0	10	NA	5.0	0.70
Sauvignon Blanc	Suvorovo	As	6.0	9.0	NA	4.0	0.60
Chardonnay	Topoli dol	As	4.0	7.0	NA	2.5	0.70
Tamyanka	Topoli dol	As	6.0	5.0	NA	2.2	2.0
Sauvignon Blanc	Brestnik	As	5.0	7.0	NA	4.7	4.0
		min	3.0	5.0	NA	2.2	0.50
		max	8.0	10.0	NA	7.2	4.0
		mean	4.8	7.5	NA	4.5	1.08
		median	5.0	7.5	NA	4.6	0.65
		st dev	1.53	1.31	NA	1.27	1.04

White varieties	Region	Element	Acetic [µg/g]	EDTA [µg/g]	Leaves [µg/g]	Must [µg/L]	Wine [µg/L]
Chardonnay 1	Oryahovo	Cd	11	17	0.130	0.110	0.070
Chardonnay 2	Oryahovo	Cd	12	20	0.040	1.50	1.20
Chardonnay 3	Oryahovo	Cd	17	21	0.110	0.250	0.150
Chardonnay 4	Oryahovo	Cd	15	18	0.080	0.090	0.040
Sauvignon Blanc	Oryahovo	Cd	12	19	0.110	0.320	0.200
Viognier	Oryahovo	Cd	6.0	21	0.040	0.080	0.020
Muscat Ottonel	Pirgovo	Cd	10	19	0.110	0.210	0.120
Chardonnay	Suvorovo	Cd	11	15	0.060	0.090	0.060
Sauvignon Blanc	Suvorovo	Cd	7.0	14	0.070	0.270	0.160
Chardonnay	Topoli dol	Cd	8.0	17	0.090	0.190	0.080
Tamyanka	Topoli dol	Cd	11	15	0.060	0.450	0.300
Sauvignon Blanc	Brestnik	Cd	10	12	0.070	6.5	5.2
		min	6.0	12	0.040	0.080	0.020
		max	17	21	0.130	6.5	5.2
		mean	11	17	0.081	0.84	0.63
		median	11	18	0.075	0.23	0.135
		st dev	3.1	2.9	0.029	1.83	1.47

White varieties	Region	Element	Acetic [µg/g]	EDTA [µg/g]	Leaves [µg/g]	Must [µg/L]	Wine [µg/L]
Chardonnay 1	Oryahovo	Co	0.010	0.010	NA	1.90	0.80
Chardonnay 2	Oryahovo	Co	0.098	0.214	NA	2.80	1.10
Chardonnay 3	Oryahovo	Co	0.045	0.068	NA	2.30	0.90
Chardonnay 4	Oryahovo	Co	0.66	0.65	NA	2.50	1.10
Sauvignon Blanc	Oryahovo	Co	0.045	0.067	NA	2.40	1.00
Viognier	Oryahovo	Co	0.53	0.074	NA	2.90	0.80
Muscat Ottonel	Pirgovo	Co	0.072	0.870	NA	2.50	1.80
Chardonnay	Suvorovo	Co	0.054	1.282	NA	3.20	1.70
Sauvignon Blanc	Suvorovo	Co	0.034	0.094	NA	2.80	1.50
Chardonnay	Topoli dol	Co	0.075	0.65	NA	2.75	1.80
Tamyanka	Topoli dol	Co	0.067	1.92	NA	2.73	1.00
Sauvignon Blanc	Brestnik	Co	0.034	0.067	NA	2.90	2.50
		min	0.010	0.010	NA	1.90	0.80
		max	0.66	1.92	NA	3.2	2.5
		mean	0.144	0.50	NA	2.6	1.33
		median	0.061	0.154	NA	2.7	1.10
		st dev	0.22	0.61	NA	0.34	0.53

White varieties	Region	Element	Acetic [µg/g]	EDTA [µg/g]	Leaves [µg/g]	Must [µg/L]	Wine [µg/L]
Chardonnay 1	Oryahovo	Cr	0.53	0.10	NA	41	30
Chardonnay 2	Oryahovo	Cr	0.30	0.26	NA	36	28
Chardonnay 3	Oryahovo	Cr	1.36	1.04	NA	32	29
Chardonnay 4	Oryahovo	Cr	2.6	0.09	NA	43	28
Sauvignon Blanc	Oryahovo	Cr	0.40	0.42	NA	54	42
Viognier	Oryahovo	Cr	1.86	0.38	NA	42	32
Muscat Ottonel	Pirgovo	Cr	0.53	0.50	NA	36	29
Chardonnay	Suvorovo	Cr	2.3	0.60	NA	54	40
Sauvignon Blanc	Suvorovo	Cr	1.00	0.84	NA	65	53
Chardonnay	Topoli dol	Cr	1.76	0.10	NA	61	51
Tamyanka	Topoli dol	Cr	1.36	0.20	NA	56	47
Sauvignon Blanc	Brestnik	Cr	1.88	0.20	NA	36	29
		min	0.30	0.09	NA	32	28
		max	2.6	1.04	NA	65	53
		mean	1.32	0.39	NA	46	36
		median	1.36	0.32	NA	43	31
		st dev	0.77	0.30	NA	11	10

White varieties	Region	Element	Acetic [µg/g]	EDTA [µg/g]	Leaves [µg/g]	Must [µg/L]	Wine [µg/L]
Chardonnay 1	Oryahovo	Li	4.3	2.5	41	16	11
Chardonnay 2	Oryahovo	Li	7.5	3.4	34	22	12
Chardonnay 3	Oryahovo	Li	5.7	2.5	36	17	10
Chardonnay 4	Oryahovo	Li	3.5	1.80	36	18	11
Sauvignon Blanc	Oryahovo	Li	6.2	2.5	24	16	12
Viognier	Oryahovo	Li	9.9	3.9	28	16	12
Muscat Ottonel	Pirgovo	Li	6.9	3.6	35	15	10
Chardonnay	Suvorovo	Li	4.4	2.8	36	8.0	5.0
Sauvignon Blanc	Suvorovo	Li	7.2	3.9	31	19	12
Chardonnay	Topoli dol	Li	1.92	2.0	18	2.5	1.9
Tamyanka	Topoli dol	Li	0.56	0.88	28	2.1	1.2
Sauvignon Blanc	Brestnik	Li	2.0	1.20	19	2.8	1.8
		min	0.56	0.88	18	2.1	1.2
		max	9.9	3.92	41	22	12
		mean	5.0	2.59	31	13	8.3
		median	5.1	2.54	33	16	11
		st dev	2.7	1.00	7.2	7.1	4.5

White varieties	Region	Element	Acetic [µg/g]	EDTA [µg/g]	Leaves [µg/g]	Must [µg/L]	Wine [µg/L]
Chardonnay 1	Oryahovo	Ni	3.1	1.68	NA	26	7.0
Chardonnay 2	Oryahovo	Ni	2.0	1.12	NA	19	10
Chardonnay 3	Oryahovo	Ni	2.6	1.05	NA	15	10
Chardonnay 4	Oryahovo	Ni	3.5	2.8	NA	31	11
Sauvignon Blanc	Oryahovo	Ni	3.4	0.87	NA	35	8.0
Viognier	Oryahovo	Ni	3.0	1.30	NA	54	5.0
Muscat Ottonel	Pirgovo	Ni	2.1	1.53	NA	31	17
Chardonnay	Suvorovo	Ni	2.7	4.8	NA	52	14
Sauvignon Blanc	Suvorovo	Ni	2.8	3.6	NA	35	12
Chardonnay	Topoli dol	Ni	2.1	1.68	NA	25	17
Tamyanka	Topoli dol	Ni	0.99	2.6	NA	36	22
Sauvignon Blanc	Brestnik	Ni	6.5	1.87	NA	32	12
		min	0.99	0.87	NA	15	5.0
		max	6.5	4.8	NA	54	22
		mean	2.9	2.1	NA	33	12
		median	2.8	1.68	NA	32	11
		st dev	1.34	1.18	NA	11	4.8

White varieties	Region	Element	Acetic [µg/g]	EDTA [µg/g]	Leaves [µg/g]	Must [µg/L]	Wine [µg/L]
Chardonnay 1	Oryahovo	Pb	0.21	2.1	0.130	13	7.2
Chardonnay 2	Oryahovo	Pb	0.160	1.8	0.160	106	66
Chardonnay 3	Oryahovo	Pb	0.92	1.0	0.130	42	28
Chardonnay 4	Oryahovo	Pb	0.160	5.5	0.25	13	10
Sauvignon Blanc	Oryahovo	Pb	0.010	1.6	0.26	18	12
Viognier	Oryahovo	Pb	0.31	1.0	0.160	7.1	4.2
Muscat Ottonel	Pirgovo	Pb	0.21	3.0	0.120	107	71
Chardonnay	Suvorovo	Pb	0.180	4.5	0.31	32	21
Sauvignon Blanc	Suvorovo	Pb	0.150	2.8	0.23	26	16
Chardonnay	Topoli dol	Pb	0.78	4.4	0.25	5.2	4.0
Tamyanka	Topoli dol	Pb	0.21	3.9	0.23	8.2	4.8
Sauvignon Blanc	Brestnik	Pb	1.10	20	1.10	130	81
		min	0.010	1.01	0.120	5.2	4.0
		max	1.10	20	1.100	130	81
		mean	0.37	4.3	0.278	42	27
		median	0.21	2.9	0.230	22	14
		st dev	0.35	5.0	0.266	45	29
