# Supplementary material for: From the Soil to the Wine—Elements’ Migration in Monovarietal Bulgarian Wines
Source: Molecules. 2025 Jan 22;30(3):475. doi: 10.3390/molecules30030475 (PMC11820015; doi:10.3390/molecules30030475)
Supplement: Supplementary file 1 [file molecules-30-00475-s001.zip › Table S5.pdf]

Table S5. Significant correlation coefficients (P<0.05).

In red wine varieties					
**macroelements**	**wine/must**	**must/acetic**	**must/EDTA**	**leaves/acetic**	**leaves/EDTA**
**Al**					
**B**	+			+	+
**Ba**				+	+
**Ca**				+	+
**Cu**					
**Fe**		+	+		
**K**	+	+	+		
**Mg**	+			+	+
**Mn**	+				
**Na**					
**P**	+				
**Sr**	+	+	+		+
**Zn**	+	+	+	+	+

**microelements**	**wine/must**	**must/acetic**	**must/EDTA**	**leaves/acetic**	**leaves/EDTA**
**As**				NA	NA
**Cd**	+		+		
**Co**				NA	NA
**Cr**	+			NA	NA
**Li**	+	+	+		
**Ni**				NA	NA
**Pb**	+	+	+	+	+

In white wine varieties					
**macroelements**	**wine/must**	**must/acetic**	**must/EDTA**	**leaves/acetic**	**leaves/EDTA**
**Al**	+				
**B**	+				
**Ba**				+	+
**Ca**					
**Cu**					
**Fe**	+				
**K**					
**Mg**					
**Mn**	+				+
**Na**					
**P**	+				
**Sr**	+	+	+	+	+
**Zn**	+			+	+

microelements	wine/must	must/acetic	must/EDTA	leaves/acetic	leaves/EDTA
**As**				NA	NA
**Cd**	+				
**Co**				NA	NA
**Cr**	+			NA	NA
**Li**	+				
**Ni**				NA	NA
**Pb**	+			+	+
